# Glymphatic influx and clearance are perturbed in Huntington’s disease

**DOI:** 10.1172/jci.insight.172286

**Published:** 2024-10-22

**Authors:** Hongshuai Liu, Lin Chen, Chuangchuang Zhang, Chang Liu, Yuguo Li, Liam Cheng, Yuxiao Ouyang, Catherine Rutledge, John Anderson, Zhiliang Wei, Ziqin Zhang, Hanzhang Lu, Peter C.M. van Zijl, Jeffrey J. Iliff, Jiadi Xu, Wenzhen Duan

**Affiliations:** 1 Division of Neurobiology, Department of Psychiatry and Behavioral Sciences, Johns Hopkins University School of Medicine, Baltimore, Maryland, USA.; 2 F.M. Kirby Research Center, Kennedy Krieger Research Institute, Baltimore, USA.; 3 Russell H. Morgan Department of Radiology and Radiological Sciences, Johns Hopkins University School of Medicine, Baltimore, Maryland, USA.; 4 Department of Biomedical Engineering, Johns Hopkins University, Baltimore, Maryland, USA.; 5 Veterans Integrated Service Network (VISN) 20 Northwest Mental Illness Research, Education, and Clinical Center (MIRECC), VA Puget Sound Health Care System, Seattle, Washington, USA.; 6 Department of Psychiatry and Behavioral Sciences and; 7 Department of Neurology, University of Washington School of Medicine, Seattle, Washington, USA.; 8 Solomon H. Snyder Department of Neuroscience, Johns Hopkins University School of Medicine, Baltimore, Maryland, USA.

**Keywords:** Neuroscience, Neurodegeneration, Neuroimaging

## Abstract

The accumulation of mutant huntingtin protein aggregates in neurons is a pathological hallmark of Huntington’s disease (HD). The glymphatic system, a brain-wide perivascular network, facilitates the exchange of interstitial fluid and cerebrospinal fluid (CSF), supporting interstitial solute clearance of brain wastes. In this study, we employed dynamic glucose-enhanced (DGE) MRI to measure d-glucose clearance from CSF as a tool to predict glymphatic function in a mouse model of HD. We found significantly diminished CSF clearance efficiency in HD mice before phenotypic onset. The impairment of CSF clearance efficiency worsened with disease progression. These DGE MRI findings in compromised glymphatic function were further verified with fluorescence-based imaging of CSF tracer influx, suggesting an impaired glymphatic function in premanifest HD. Moreover, expression of the astroglial water channel aquaporin-4 in the perivascular compartment, a key mediator of glymphatic function, was significantly diminished in both HD mouse brain and human HD brain. Our data, acquired using a clinically translatable MRI, indicate a perturbed glymphatic network in the HD brain. Further validation of these findings in clinical studies will provide insights into the potential of glymphatic clearance as a therapeutic target as well as an early biomarker in HD.

## Abstract 2

Impaired glymphatic clearance function is evident in premanifest Huntington’s disease mice

## Graphical abstract

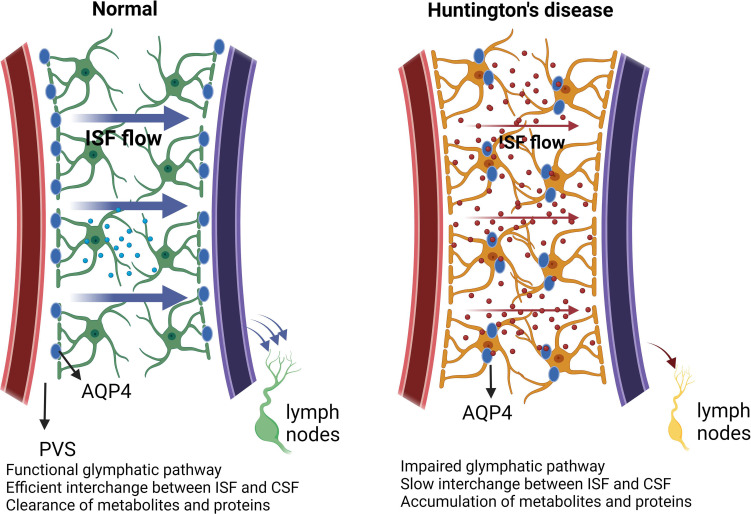

## Introduction

Huntington’s disease (HD) is a dominantly inherited, fatal neurodegenerative disorder caused by a CAG expansion in exon 1 of the *Huntingtin* (*HTT*) gene, which leads to the production of a toxic mutant huntingtin protein (mHTT) (1). A fundamental pathological hallmark of HD is abnormal mHTT accumulation in the brain, where the misfolded and aggregated disease-causative protein propagates and spreads in a prion-like fashion (2).

The principle of brain homeostasis asserts that the elimination of proteins must be in equilibrium with their synthesis, utilization, metabolism, and localized degradation. Interstitial solutes, including proteins, can be cleared across the blood-brain barrier and undergo local cellular uptake and degradation. When not subject to these cellular processes, it was generally believed that they were cleared through exchange with the surrounding cerebrospinal fluid (CSF) in a process that was considered slow and diffuse. Beginning in 2012, the glymphatic (glial-lymphatic) model of fluid and solute exchange was described (3), in which CSF and interstitial fluid (ISF) and solute exchange were observed to be rapid, anatomically organized along perivascular spaces surrounding the cerebral vasculature, and regulated by the sleep-wake cycle (4). More recently, meningeal lymphatic vessels associated with dural sinuses have been characterized that contribute to the clearance of solutes, including those arising from the brain interstitium and from the CSF compartment (5–7). The combined activity of perivascular glymphatic exchange and meningeal lymphatic clearance appears to function together to subserve brain interstitial solute and waste clearance. Importantly, key features of these processes, including perivascular CSF-ISF solute exchange, sleep-active brain solute clearance, and parasagittal solute uptake, have been confirmed in the human brain (8).

The glymphatic system is composed of meningeal lymphatic vessels that drain CSF and ISF toward cervical lymph nodes (4). The function of the glymphatic system is supported by the aquaporin-4 (AQP4) water channel, which presents with high density in perivascular astrocytic endfoot membranes (4). Glymphatic function is a highly regulated process, with changes in its activity accompanying aging as well as disease conditions (9–11). The efficiency of glymphatic clearance is lowered when AQP4 perivascular localization is reduced (12, 13). There is a growing awareness that such reduced AQP4 perivascular localization occurs in neurological disorders (14–17), which in turn is associated with the expression of a protein complex including α-syntrophin (SNTA1) (18, 19).

Evidence has begun to emerge that failure of the glymphatic system leads to an increase of local mHTT concentrations to levels that favor aggregation. One recent study suggested that a dysfunctional glymphatic system may contribute to HD manifestation and interrupt antisense oligonucleotide (ASO) distribution throughout the entire brain (20). Another study reports that secretion of mHTT from cells in the brain, followed by glymphatic clearance from the extracellular space, contributes to mHTT in the CSF (21). Whether the glymphatic system is disturbed in the HD brain, particularly in the premanifest stage, remains unclear.

In this study, we combined an in vivo measure of CSF clearance capacity by dynamic glucose-enhanced (DGE) MRI (22) with fluorescence-based imaging of glymphatic CSF tracer influx in a widely used zQ175-knockin HD mouse model. We observed that CSF clearance efficiency and glymphatic function were impaired in the zQ175 HD mice, before the manifestation of motor deficits and MRI detection of striatal atrophy. The impairment of CSF clearance worsened along with HD progression. Further mechanistic study indicated that AQP4 perivascular localization, a key contributor to glymphatic function, was significantly reduced in both HD mice and HD human brains. These findings set the premise to further investigate the role of glymphatic function in HD pathogenesis as a potential therapeutic target as well as an early biomarker for this devastating disease.

## Results

### CSF clearance capacity measured by DGE MRI is Aqp4 dependent and reflects glymphatic CSF-ISF exchange capacity in mice.

We recently developed a DGE MRI (22–25) approach that can sensitively assess d-glucose signal changes in the mouse CSF (26). Because glucose transporters are highly enriched in the brain capillary endothelial cells and choroid plexus epithelial cells (27, 28), d-glucose can rapidly penetrate the blood CSF barrier (BCSFB) and enter CSF. This unique feature provides an opportunity to measure CSF clearance efficiency by monitoring d-glucose levels in CSF following intravenous delivery. Because AQP4 (protein) is essential for functional glymphatic transport (29) and significantly compromised glymphatic function has been reported in *Aqp4*-KO mice (30), we first evaluated CSF clearance efficiency in an *Aqp4*-KO mouse model.

The absence of Aqp4 expression in the *Aqp4*-KO mice was verified (Supplemental Figure 1A; supplemental material available online with this article; https://doi.org/10.1172/jci.insight.172286DS1); we then conducted DGE MRI scans in both 4-month-old *Aqp4*-KO mice and wild-type (WT) mice. Following d-glucose injection via the tail vein, DGE signal in the CSF rapidly reached a peak in both mouse genotypes. In WT mice, d-glucose signal decayed gradually following the peak, indicating gradual d-glucose clearance from CSF, while DGE signal in the CSF of *Aqp4*-KO mice was sustained or even slightly elevated during the MRI scan session (40 minutes) (Supplemental Figure 1, B and C). The uptake of d-glucose in CSF was comparable (*P* > 0.05) in the 2 groups (Supplemental Figure 1D), while d-glucose clearance was significantly lower (*P* < 0.05) in *Aqp4*-KO mice than WT mice (Supplemental Figure 1E). Here we used a γ-variate model with 3 unknowns (S_max_, μ_in_, and μ_out_) to fit the uptake and get an initial rate estimate (μ_out_) for the clearance by fitting only the first 20 minutes after infusion. This method from our previous studies (26) is summarized in the Supplemental Methods. Taken together, these results suggest that loss of the *Aqp4* gene, which suppresses perivascular glymphatic exchange, slows CSF d-glucose clearance in mice. Thus, CSF d-glucose clearance capacity is AQP4 dependent.

In subsequent experiments (detailed below), we evaluated whether altered CSF clearance efficiency paralleled changes in the influx of fluorescent CSF tracers into the brain tissue of HD mice, a validated approach for assessing glymphatic function in rodents. Importantly, similar results were observed. Combined, these results strongly support the notion that CSF d-glucose clearance efficiency reflects glymphatic CSF-ISF exchange capacity.

### Impaired glymphatic function is evident in zQ175 HD mice before phenotypic onset from measures of (i) CSF clearance efficiency by DGE MRI and (ii) influx of a fluorescence CSF tracer from cisterna magna to brain parenchyma.

The zQ175 knockin model has been well characterized and used in HD preclinical studies. Heterozygous zQ175 HD mice start to show significant striatal atrophy and motor deficits on the balance beam around 6 months of age, with symptoms progressing along with age. Using structural MRI measures and motor function assessment, we verified that there was no significant striatal atrophy (Supplemental Figure 2A), and motor function on the balance beam (Supplemental Figure 2B) was preserved in 4-month-old zQ175 (heterozygous) HD mice, indicating that this age is a premanifest stage of zQ175 HD model.

**Figure 1:**
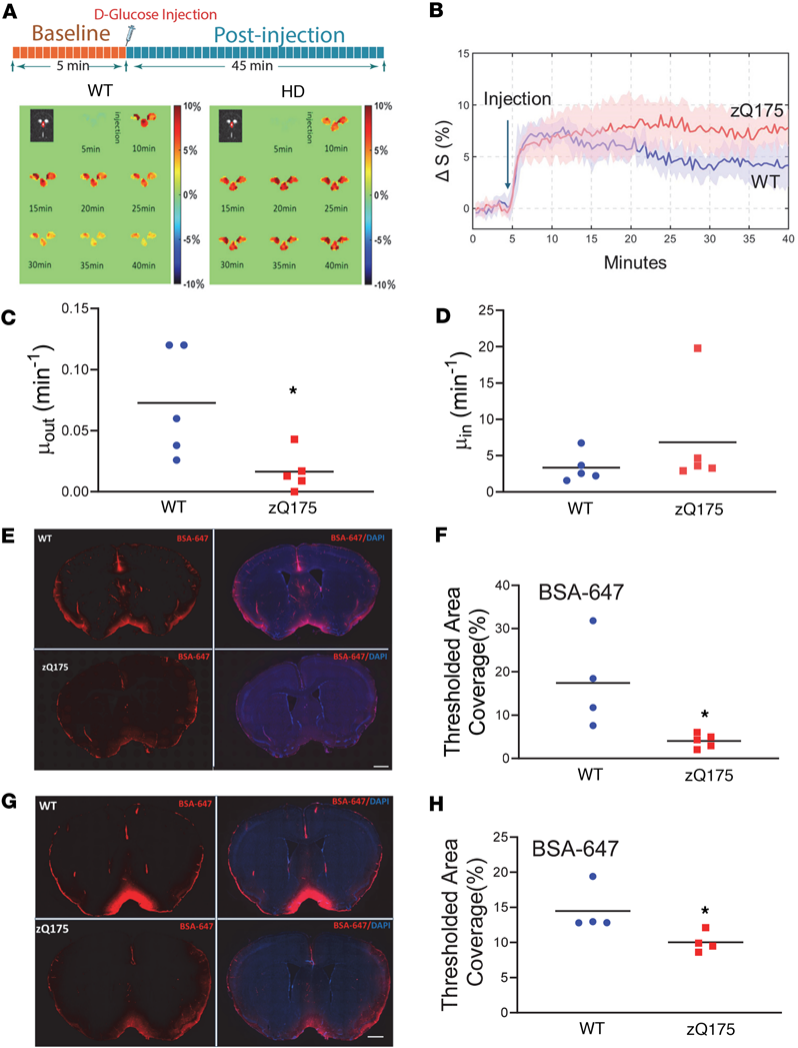
Impaired glymphatic system as revealed by DGE MRI and fluorescence-based imaging in premanifest zQ175 HD mice. (**A**) Illustration of DGE MRI scan timeline (upper panel) and representative DGE images for the third ventricle (lower panel) in a WT mouse and a zQ175 mouse at 4 months of age. (**B**) Average CSF-based DGE signal changes during the entire scan period from male WT and zQ175 mice. *n* = 5 mice/genotype. (**C**) Comparison of fitted clearance parameter μ_out_ for CSF. *P < 0.05 vs. WT by standard Student’s *t* test. (**D**) Comparison of fitted uptake parameter μ_in_ for CSF. (**E**) Representative images of BSA-647 fluorescent dye distribution in the brain parenchyma at 60 minutes after intra-CM injection. Note the wide distribution of fluorescent dye along the glymphatic pathway in WT mice, while very limited fluorescence distribution was seen in the HD mouse brain. Scale bar = 1 cm. The left panel shows the BSA-647 fluorescence images, and the right panel shows BSA-647 fluorescence images merged with DAPI staining images. (**F**) Quantification of the fluorescent dye distribution at 60 minutes after CSF tracer injection. *P < 0.05 vs. WT by standard Student’s *t* test. (**G**) Representative images of BSA-647 fluorescent dye distribution in the brain parenchyma at 180 minutes after intra-CM injection. Scale bar = 1 cm. (**H**) Quantification of the fluorescent dye distribution at 180 minutes after tracer injection. *P < 0.05 vs. WT by standard Student’s *t* test.

We then used DGE MRI to assess CSF d-glucose clearance in 4-month-old zQ175 HD mice. After a baseline DGE signal was established, d-glucose was injected via the tail vein into 4-month-old zQ175 mice and WT littermate controls, and DGE MRI scans were conducted over a 40-minute period. We monitored DGE signals in the CSF of the third ventricle (representative images shown in Figure 1A). DGE signals reached a peak in the CSF within 1–2 minutes following i.v. injection of d-glucose. WT mice showed a gradual DGE signal decay, indicating normal d-glucose clearance in the CSF. In contrast, zQ175 HD mice exhibited sustained d-glucose signals in the CSF for the entire scan period (Figure 1, A and B) (*P* < 0.05 vs. WT). The d-glucose clearance in zQ175 HD mice was significantly slower than that in WT mice (*P* < 0.05, Figure 1C), while the d-glucose uptake in CSF was comparable between groups (*P* < 0.05, Figure 1D). These CSF d-glucose uptake results were consistent with the levels of glucose transporters in the brain (Supplemental Figure 2C).

To determine whether blood d-glucose levels influenced d-glucose uptake/clearance in the CSF, dynamic glucose signals in the sagittal sinus were monitored during the entire scan period. We observed no significant difference in blood DGE signals between the 2 genotypes (Supplemental Figure 2E), including glucose clearance, uptake, and maximal d-glucose signal difference in the vein (Supplemental Figure 2, F–H). These results indicate that the CSF data from Figure 1 suggest a potential impairment of glymphatic function in premanifest zQ175 HD mice.

We further validated these MRI findings with a gold standard, fluorescence-based CSF tracer imaging technique. A fluorescent tracer, Alexa Fluor 647–labeled BSA (BSA-647), was injected into the cisterna magna (CM) in premanifest zQ175 mice to evaluate the glymphatic influx efficiency; 60 minutes and 180 minutes following tracer injection, BSA-647 fluorescent dye distribution in the parenchyma was quantified. We observed significantly reduced fluorescent tracer movement into the brain parenchyma along the glymphatic network in 4-month-old zQ175 HD mice compared with age-matched WT controls at both time points (Figure 1, E–H), verifying the reduced glymphatic influx in premanifest zQ175 HD mice. Furthermore, we injected BSA-647 into the striatum to evaluate glymphatic efflux efficiency in zQ175 mice. At 180 minutes after the tracer injection, we observed a trend of more tracers sustained in the striatum in zQ175 mice compared with those in the littermate control mice (Supplemental Figure 3A) but no statistical difference (Supplemental Figure 3B). Taken together, these data demonstrate the capacity of DGE MRI to detect glymphatic function and that glymphatic function, particularly glymphatic influx, is compromised in zQ175 HD mice before striatal atrophy and motor deficits.

### Reduced Aqp4 perivascular localization in premanifest zQ175 HD mouse brain.

**Figure 2:**
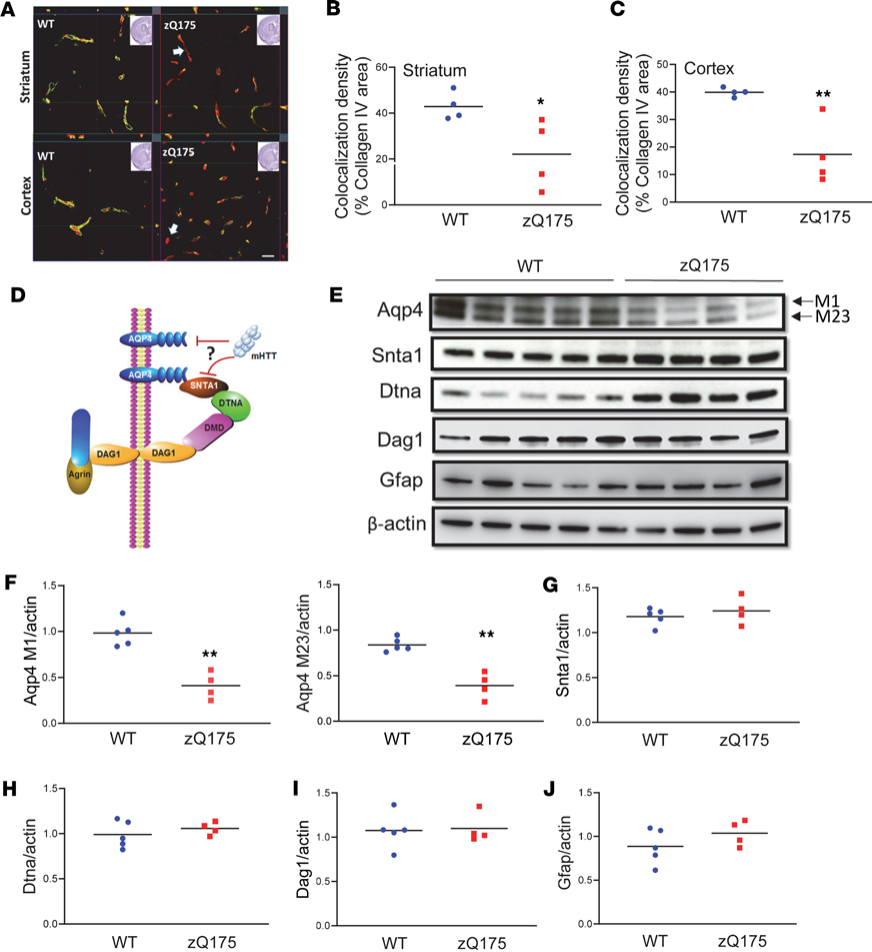
Decreased AQP4 perivascular localization (polarization) and levels in the brain of premanifest zQ175 HD mice. (**A**) Representative *Z*-stack images of coimmunofluorescence staining of AQP4 and collagen IV in the striatum and cortex of 4-month-old zQ175 and WT controls. Scale bar = 100 μm. (**B** and **C**) Quantification of colocalized pixels of AQP4 (green in **A**) and collagen IV (red in **A**) in the striatum (**B**) and cortex (**C**) of 4-month-old male zQ175 mice and WT controls. *P < 0.05, **P < 0.01 vs. WT by standard Student’s *t* test. (**D**) Illustration of AQP4 and its astrocytic endfeet–anchoring protein complex. (**E**–**J**) Western blots (**E**) and quantification of Aqp4 (**F**); Aqp4-anchoring protein complex components Snta1 (**G**), Dtna (**H**), and Dag1 (**I**); and activated astrocyte marker Gfap (**J**). **P < 0.01 vs. WT by standard Student’s *t* test. Aqp4, aquaporin-4; Snta1, syntrophin alpha 1; Dtna, dystrobrevin alpha; Dag1, dystroglycan 1; Gfap, glial fibrillary acidic protein.

Glymphatic clearance efficiency is mediated by astrocytic AQP4 water channels that are present at high density in perivascular astrocytic endfoot membranes. Thus, AQP4-dependent convective flow is critical for effective glymphatic clearance, and AQP4 perivascular localization is required for glymphatic function. To determine if Aqp4 perivascular localization was altered in the 4-month-old zQ175 HD mouse brain, we performed coimmunostaining of Aqp4 and vascular marker protein collagen IV to examine Aqp4 perivascular colocalization. As visualized, Aqp4 immunosignals were concentrated on the perivascular domains in both the striatum and cerebral cortex (Figure 2, A–C) of WT mice, indicated by colocalized Aqp4 and collagen IV immunosignals. In contrast, zQ175 HD mice exhibited significantly reduced Aqp4 immunosignals in the perivascular domains in both the striatum (Figure 2, A and B) and cerebral cortex (Figure 2, A and C), suggesting loss of AQP4 perivascular localization in the zQ175 HD mouse brain before phenotypic onset.

AQP4 polarization requires the presence of a functional protein complex formed by a key protein, SNTA1, which connects AQP4 with other complex proteins, dystrobrevin-α (DTNA) and dystroglycan (DAG1) (30) (Figure 2D), and this protein complex anchors AQP4 to the astrocytic endfeet perivascular location. The absence of the key component protein SNTA1 interferes with AQP4 perivascular localization (18), which may disrupt glymphatic function. To determine whether loss of Aqp4 perivascular localization was due to reduced levels of Aqp4 or its astrocytic endfeet–anchoring machinery, we examined protein levels of Aqp4, Snta1, Dtna, and Dag1 in the striatum of 4-month-old zQ175 mice (Figure 2E). There are 2 major isoforms of Aqp4 in the mouse brain, M1 and M23. We found that both isoforms of Aqp4 protein were significantly (*P* < 0.01) reduced in the premanifest zQ175 mouse striatum (Figure 2F). There was no significant difference in the protein levels of all 3 Aqp4-anchoring proteins at this age (Figure 2, G–I). Because Aqp4 is preferentially expressed in astrocytes in the brain, we also examined Gfap levels, and our results indicated no detectable changes in Gfap levels (Figure 2J). These data suggest that the loss of Aqp4 perivascular localization in 4-month-old zQ175 HD mice is mainly due to reduced Aqp4 protein levels.

### Perturbed glymphatic function and reduced Aqp4 perivascular localization in manifest zQ175 HD mice.

**Figure 3:**
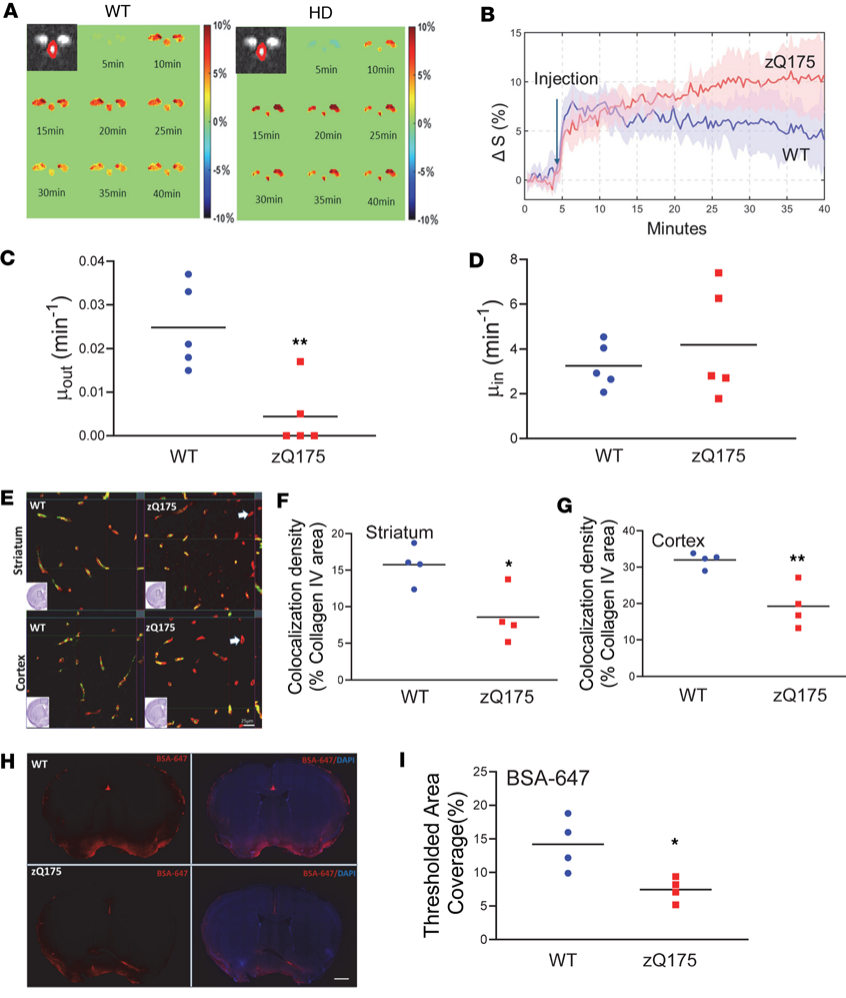
Perturbed glymphatic function and exacerbated AQP4 perivascular localization (polarization) in the manifest zQ175 HD mouse brain. (**A**) Representative images of DGE signals (lower panel) in a WT mouse and a zQ175 mouse at 10 months of age. (**B**) Average dynamic d-glucose signals during the entire scan period from male WT and zQ75 mice. *n* = 5 mice/genotype. (**C**) Comparison of fitted clearance parameter μ_out_ d-glucose clearance rate in CSF of the third ventricle. **P < 0.01 vs. WT by standard Student’s *t* test. (**D**) Comparison of fitted clearance parameter μ_in_ d-glucose uptake rate in CSF of the third ventricle. (**E**) Representative images of coimmunofluorescence staining of AQP4 and collagen IV in the striatum (upper panel) and cortex (bottom panel) of 10-month-old male zQ175 and WT controls. Scale bar = 100 μm. (**F**) Quantification of colocalized pixels of AQP4 (green in **E**) and collagen IV (red in **E**) in the striatum of 10-month-old male zQ175 mice and WT controls. *P < 0.05 vs. WT by standard Student’s *t* test. (**G**) Quantification of colocalized pixels of AQP4 (green in **E**) and collagen IV (red in **E**) in the cortex of 10-month-old male zQ175 mice and WT controls. **P < 0.01 vs. WT by standard Student’s *t* test. (**H**) Representative images of BSA-647 fluorescent dye distribution in the brain parenchyma at 60 minutes after intra-CM injection in 10-month-old mice. Scale bar = 1 cm. The left panel shows the BSA-647 fluorescence images, and the right panel shows BSA-647 fluorescence images merged with DAPI staining images. (**I**) Quantification of the fluorescent dye distribution at 60 minutes after CSF tracer injection. *P < 0.05 vs. WT by standard Student’s *t* test.

Like patients with HD, zQ175 HD mice progressively develop into the symptomatic (manifest) stage, displaying motor deficits and striatal atrophy (31, 32). Using structural MRI measures and motor testing on a balance beam, we verified that 10-month-old zQ175 HD mice exhibited significant (*P* < 0.05, 1-tailed *t* test) striatal atrophy (Supplemental Figure 5A) and motor deficits, indicated by prolonged traverse time (*P* < 0.05) on the beam compared with their age-matched WT control mice (Supplemental Figure 5B). We then sought to determine whether glymphatic dysfunction progresses along with disease progression in zQ175 mice. We conducted DGE MRI scans on 10-month-old zQ175 mice. zQ175 mice exhibited slower DGE signal decay in the CSF during an entire scan period, while WT mice still performed well in the CSF clearance of d-glucose (Figure 3, A and B). Further imaging analysis indicated that CSF d-glucose clearance was significantly slower (*P* < 0.01) in zQ175 than in WT mice (Figure 3C), while CSF d-glucose uptake had no significant difference between the 2 genotypes (Figure 3D). These data suggest that manifest zQ175 HD mice have exacerbated disruption of glymphatic function. By comparison of 4-month-old and 10-month-old data, we also noticed an age-dependent decline in glymphatic function in control mice (Supplemental Figure 4).

We then asked whether d-glucose uptake in the parenchyma of manifest zQ175 HD mice was altered. Western blotting analysis of glucose transporter 1 (Glut1) and 3 (Glut3) in the striatum indicated that there were no significant changes in levels of Glut1, which is a major glucose transporter expressed in glia and endothelial/epithelial cells (Supplemental Figure 5, C and D). Levels of Glut3, which is preferentially expressed in neurons, were decreased in the manifest zQ175 mouse striatum (*P* < 0.01, Supplemental Figure 5, C and E). Consistent with Western blot findings, we observed significantly lower d-glucose uptake in the striatum of manifest zQ175 mice compared with age-matched controls (*P* < 0.05, Supplemental Figure 5, F–H). Taken together, reduced d-glucose uptake in the zQ175 HD mouse striatum is likely due to reduced neuronal Glut3 levels. No changes in Glut1 protein levels are consistent with no changes in d-glucose uptake in the CSF.

Given the critical role astrocytic Aqp4 perivascular localization plays in the control of glymphatic function, we performed coimmunostaining with antibodies against Aqp4 and collagen IV. Supporting our imaging findings of impaired glymphatic function detected by DGE MRI, Aqp4 perivascular localization in the manifest zQ175 mouse brain was significantly diminished, indicated by decreased colocalization of Aqp4 and collagen IV in the striatum (*P* < 0.05, Figure 3, E and F) and cortex (*P* < 0.01, Figure 3, E and G). Furthermore, we validated the MRI findings with a fluorescence-based CSF tracer imaging technique. Fluorescent tracer BSA-647 was injected into the CM in 10-month-old zQ175 mice or WT littermate controls to evaluate glymphatic influx efficiency; 60 minutes following tracer injection, BSA-647 fluorescent dye distribution in the parenchyma was quantified. We observed significantly reduced fluorescent tracer movement into the brain parenchyma along the glymphatic network in 10-month-old zQ175 HD mice compared with age-matched WT controls (Figure 3, H and I).

To dissect the underlying molecular basis of reduced Aqp4 perivascular localization in manifest zQ175 HD mice, we quantified the levels of Aqp4 and its astrocytic endfeet–anchoring protein Snta1 in the mouse striatum at 10 months of age. When we quantified levels of these proteins using a ratio to β-actin, we found that Aqp4 M1, M23, and Snta1 levels had no significant difference between HD mice and age-matched controls (Supplemental Figure 6, A and B). Meanwhile, we noticed increased Gfap protein levels (Supplemental Figure 6, A and B) and Gfap-positive cells (Supplemental Figure 7, A and B) in the zQ175 HD mice at the manifest age, indicating astrogliosis in the HD mouse brain. Taken together, our data suggest that reduced expression of Aqp4 on the perivascular astrocyte endfeet (polarization) rather than Aqp4 protein levels may contribute to compromised glymphatic function in the manifest zQ175 mice.

### Reduced AQP4 perivascular polarization is evident in human HD brain.

**Figure 4:**
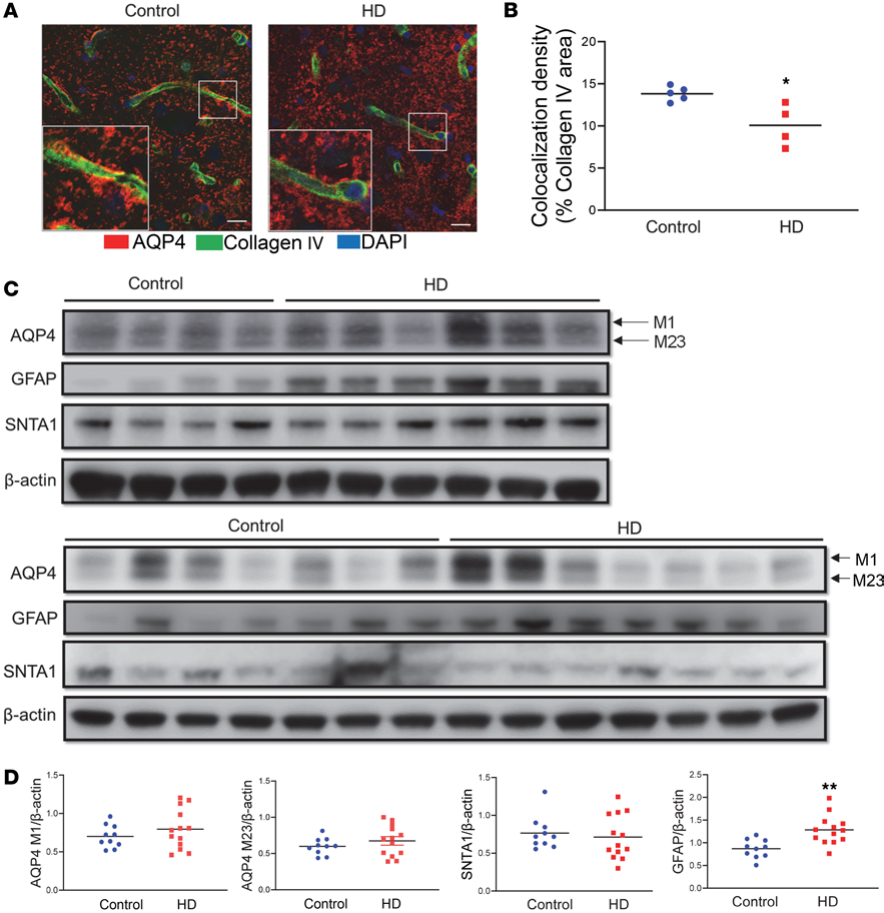
Reduced perivascular AQP4 localization accompanying astrogliosis in the human HD brain. (**A**) Representative images of coimmunofluorescence staining of AQP4 (red) and collagen IV (green) in the caudate putamen of a patient with HD and age-matched control. Scale bar = 100 μm. Insets in **A** are 3 times enlarged from the original images. (**B**) Quantification of colocalized pixels of AQP4 (red in **A**) and collagen IV (green in **A**) in the caudate putamen of HD patients (*n* = 6) and age-matched controls (*n* = 4). *P < 0.05 vs. control by standard Student’s *t* test. (**C**) Western blots of AQP4, SNTA1, and GFAP in the human caudate samples from 13 HD brains and 10 control brains. (**D**) Quantification of AQP4 (both isoforms), SNTA1, and GFAP protein levels (ratio to the loading control β-actin) in the caudate samples. **P < 0.01 vs. control by standard Student’s *t* test.

The next key question is whether a compromised glymphatic network occurs in the human HD brain. We obtained postmortem caudate samples from NIH NeuroBioBank (Supplemental Table 1 — fixed tissue samples for immunostaining; Supplemental Table 2 — frozen caudate samples for Western blotting). Our coimmunostaining results with antibodies against AQP4 and collagen IV revealed reduced AQP4 immunosignals in perivascular areas (Figure 4A); the quantification of colocalized immunosignals of AQP4 and collagen IV illustrated significantly reduced AQP4 perivascular localization in human HD brains (*P* < 0.05, Figure 4B). We then examined protein levels of AQP4, SNTA1, and GFAP in 10 control and 13 HD caudate samples. These human HD caudate samples originated from patients with Vonsattel Grade 2 and Grade 3 (see details in Supplemental Table 2). We observed increased astrogliosis (increased GFAP levels) in all HD caudate samples (Figure 4, C and D). There was greater variability observed in the levels of the AQP4 isoforms, M1 and M23, and SNTA1 in human samples. However, no statistically significant differences were found between the control group and patients with HD for these proteins (Figure 4, C and D). These findings in human samples are consistent with those from manifest zQ175 mice, which showed a significant reduction in the expression of polarized AQP4 rather than changes in overall protein levels in manifest HD.

## Discussion

In this study, we first assessed glymphatic function with DGE MRI and then validated the findings with gold standard fluorescent CSF tracer influx measures in a widely used HD knockin mouse model. Our results demonstrate that zQ175 HD mice exhibited substantially impaired glymphatic function before detectable striatal atrophy and motor deficits. A mechanistic experiment suggests that decreased AQP4 levels and its perivascular localization underlie the impaired glymphatic network in premanifest zQ175 HD mice. In both manifest HD mice and human HD brains, reduced expression of polarized AQP4 rather than AQP4 protein levels mainly contribute to the perturbed glymphatic function, and astrogliosis may worsen already compromised glymphatic clearance capacity in manifest HD.

A fundamental problem in HD and other neurodegenerative disorders is abnormal protein accumulation in the brain, where misfolded and aggregated disease-causative proteins can propagate and spread in a prion-like fashion (2). Here we used a recently developed DGE MRI approach, with i.v. d-glucose as a natural biodegradable tracer, to investigate glymphatic function in an HD mouse model. The data show that the glymphatic network is impaired in premanifest HD mice and worsens in manifest HD. Our DGE MRI findings on impairment of glymphatic function in HD mice were further validated by fluorescence-based imaging of CSF tracer distribution in the parenchyma (3). Although a recent study suggests that a dysfunctional glymphatic system may interrupt ASO brain distribution (20), HD has been far less studied in the context of glymphatic function. Our present work advances the understanding of a brain-wide waste clearance pathway that is functionally interconnected with the brain neurovascular system and its contribution to HD pathogenesis. In the long run, our data provide fundamental insights into the behavior of the glymphatic system in HD and a mechanistic basis for identifying new targets for disease-modifying treatment for HD.

Evidence is being built that failure of the glymphatic system leads to an increase of local protein concentrations to levels that favor aggregation. The glymphatic system is a highly organized CSF-ISF transport system with several key functions, including exporting excess metabolite/protein wastes in ISF from brains. The glymphatic system’s perivascular pathways are directly connected to the subarachnoid spaces surrounding the brain, from which CSF is rapidly driven into deep regions of the brain. Regardless of its precise efflux pathways, CSF ultimately drains into the cervical lymphatic vasculature. Caron et al. reported that mHTT is present in the CSF of HD mice in the absence of neurodegeneration (21). More specifically, their study showed that secretion of mHTT from cells in the CNS, followed by glymphatic clearance from the extracellular space, contributes to mHTT in the CSF (21). The discovery that disease-relevant misfolded and aggregated proteins, such as mHTT, α-synuclein, and amyloid-β (Aβ), can propagate and spread in a prion-like fashion has sparked considerable interest (2). It has been postulated that seeding occurs across brain regions that are synaptically connected. However, the evidence for synaptic spread is largely based on post hoc analysis of anatomic networks; it remains unclear how synaptic relationships by themselves can mediate seeding. It is possible that aggregates simply spread via the extracellular spaces of the glymphatic network and that aging- or disease-relevant gene mutations deteriorate the glymphatic network, which could slow the clearance of toxic protein waste. This hypothesis was evident in a mouse Alzheimer’s disease (AD) model, in which deletion of *Aqp4* sharply increased both Aβ plaque formation and cognitive deficits (33). It might also be the case in HD, though further examination is needed to confirm this hypothesis.

Glymphatic function is a highly regulated process, with changes in its activity accompanying aging as well as disease conditions (9–11). The efficiency of glymphatic clearance is diminished by reduced AQP4 perivascular localization (12, 13). There is a growing awareness that reduced perivascular AQP4 occurs in pathological conditions (14–17). Deleting *AQP4* water channels reduced CSF tracer movement from the periarterial spaces into the interstitium and resulted in markedly impaired clearance of extracellular solutes (34). In our study, we detected impaired d-glucose clearance in the CSF of *Aqp4*-KO mice, and we further observed dramatically decreased Aqp4 levels and its perivascular localization in the premanifest HD mouse brain, which underlies perturbation of glymphatic function. Theories explaining the drainage of brain metabolites via different extracerebral glymphatic pathways are revised now in light of novel findings, including the function of meningeal lymphatics (6, 7). Recent data suggest that the glymphatic system and meningeal lymphatic vessels drain macromolecules from the brain parenchyma to the deep cervical lymph nodes (6, 35).

The brain is an energy-demanding organ that weighs ~2% of the whole body but consumes ~20% of total body d-glucose in the resting state (36). Strong evidence of early d-glucose hypometabolism in the HD brain has been reported (37, 38). A decreased expression of glucose transporters GLUT1 and GLUT3 was also reported in postmortem HD brains (39). However, within the brain, when and how many glucose transporters and metabolic changes occur during disease progression remain controversial. Uptake and metabolism of d-glucose may appear to function differently at distinct disease stages in HD. Whether altered d-glucose uptake and metabolism in premanifest HD represent irreversible damage or compensatory change remains unknown. We found d-glucose uptake was decreased in the manifest zQ175 mouse striatum but not in CSF, which is consistent with decreased Glut3 levels but not Glut1 levels in the striatum of these HD mice.

The high metabolic rate of the brain requires rapid infusion and clearance of metabolic products because neurons and glial cells are very sensitive to changes in their extracellular homeostasis. The glymphatic system facilitates the convective exchange of various interstitial solutes between CSF and ISF and is important for the brain-wide delivery of nutrients, specifically d-glucose (40–42). Taken together, DGE MRI can measure dynamic d-glucose clearance in the CSF, which has the potential to serve as a biomarker of brain glymphatic function. The decline of glymphatic function in premanifest HD condition suggests that treatments directed at restoring normal glymphatic transport and waste removal from the brain may become a preventive or therapeutic approach for this devastating disease.

## Methods

Further information can be found in Supplemental Methods.

### Sex as a biological variable.

The DGE MRI scans were conducted in male mice because male mice exhibited less variability in phenotypes. The findings are expected to be relevant to more than one sex.

### Animals.

Heterozygous zQ175 mice and WT littermates were used. zQ175 breeders were obtained from the Jackson Laboratory. Genotyping and CAG repeat size were determined by PCR of tail snips at Laragen Inc. The CAG repeat length of zQ175 mice used in the study was 220 ± 3. All mice were housed under specific pathogen–free conditions with a reversed 12-hour light/12-hour dark cycle maintained at 23°C and provided food and water ad libitum. All behavioral tests and MRI measures were done in the mouse dark phase (active). During MRI scanning, mice were under isoflurane anesthesia to minimize suffering (see below for details). Power analyses were used to determine the required number of mice per group. To prevent bias, mice underwent randomization into groups. Data were collected using unique animal IDs and analyzed by investigators masked to the genotype. Outliers were not excluded unless there were rare occurrences, such as unusual artifacts or animal death, that impeded data acquisition. Each experimental group comprised a mixture of multiple litters from different dams and sires. In comparisons driven by genotype, littermate controls were utilized. To minimize bias, experimenters remained masked to genotype/treatment throughout the studies and during the quantification process. Data were collected using animal ID and analyzed by investigators who were masked to genotype and treatment.

### MRI acquisition.

In vivo MRI experiments were performed on a horizontal-bore 11.7 T Bruker Biospec system equipped with a 72 mm quadrature transmitter coil and a 4-element (2 × 2) phased-array receiver coil. The MRI detection used an onVDMP sequence (22) consisting of a train of binomial pulses; each pulse shape was 2 block pulses of opposite phases with a width of 1.5 ms. Peak B1 strength was 15.6 μT. The flip angle of each block pulse was 360°. The offset of the binomial pulses was set to the water resonance, and the mixing time between pulse pairs was set to 10 ms to remove unwanted traverse magnetization. After the labeling module, anatomical images covering the third ventricle were acquired using a multislice rapid acquisition with relaxation enhancement (RARE) sequence: resolution = 0.1 × 0.1 × 0.5 mm^3^, echo time = 3 ms, repetition time = 2.5 seconds, mixing time = 10 ms, average number = 128, RARE factor = 23, slice thickness = 1 mm, a matrix size of 196 × 128, and a field of view of 20 × 10 mm^2^. A multislice approach was used to observe glucose uptake in both the striatum and CSF. DGE images were acquired continuously for 50 minutes. A 5-minute prescan was performed before glucose infusion as a dummy scan to ensure the mouse was stabilized in the scanner, and then a bolus of 0.15 mL 50% w/w d-glucose (0.5 g/mL, clinical-grade dextrose, Hospira) was infused (rate, 0.15 mL/min) through the tail vein using a syringe pump (Harvard Apparatus). The amount of glucose for each mouse depended on body weight (6 μL/g).

All mice were anesthetized using 2% isoflurane and maintained by 1%–1.5% isoflurane during the MRI scan. The mouse was placed on a water-heated bed equipped with a pressure sensor. The animal’s head was positioned with a bite bar and a container. The respiratory rate of the mouse was monitored (SAII) and maintained at 40–60 breaths/min.

### DGE MRI with onVDMP sequence image analysis.

Statistical parametric mapping (Version 8, Wellcome Trust Centre for Neuroimaging, London, United Kingdom; http://www.fil.ion.ucl.ac.uk/spm/) and in-house programs coded in MATLAB (MathWorks) were used. DGE MRI was measured with the onVDMP pulse sequence consisting of a train of binomial pulses composed of 2 high-power pulses with alternating phase (*pp*) (22). The binomial pulse is designed to label all exchanging protons in a way that depends on the excitation profile, designed to avoid water resonance. Motion correction between DGE images was performed with Medical Imaging Registration Toolbox, and the difference in signal was quantified with the surround subtraction method. Chemical Exchange Saturation Transfer (CEST) approaches such as onVDMP MRI can also be described using T1ρ theory, an MRI technique that measures the spin-lattice relaxation time constant in a rotating magnetic field. The baseline signal and curve fitting follow our previous publications (26, 41) on DGE MRI. Two regions of interest were manually selected in each scan: 1) CSF (the third ventricle) and 2) striatum. The measurement of time-resolved changes of DGE MRI signal after an i.v. bolus infusion of d-glucose reports changes in glucose concentrations in biological tissues and thus contains information on d-glucose delivery, transport, metabolism, as well as clearance kinetics.

This molecular MRI method allows high labeling efficiency by utilizing a train of short pulses leading to an improved CEST signal for glucose hydroxyl protons compared with conventional CEST MRI. To facilitate the translation of this DGE MRI to clinical use, we tested the onVDMP pulse sequence to detect d-glucose CSF clearance on a 3 T scanner, a common clinical MRI field strength (26). We also showed the feasibility of this modification in other neurodegenerative disorders, including 2 mouse models of AD (26, 43). Glucose transporters are highly enriched in brain capillary endothelial cells; thus, d-glucose can easily penetrate the BCSFB and enter CSF. This provides an opportunity to monitor the CSF-ISF exchange process through the i.v. administration of d-glucose, a minimally invasive method that is a routine clinical practice.

### Intracisternal tracer injection.

Anesthetized mice with the same concentration of isoflurane used in MRI were fixed in a stereotaxic frame, the CM was surgically exposed, and a 30G needle was inserted into the CM. The fluorescent CSF tracer (BSA, Alexa Fluor 647 conjugate, Invitrogen, Thermo Fisher Scientific; 66 kDa) was dissolved in artificial CSF at a concentration of 0.5% (w/v). A total of 10 μL of CSF tracer was injected at a rate of 2 μL/min for 5 minutes using a syringe pump (Harvard Apparatus). All experiments were conducted by the same operator. To visualize tracer movement from the subarachnoid space into the brain parenchyma, mice were perfusion-fixed 1 hour after intracisternal tracer injection. Coronal vibratome slices, 100 μm, were cut and mounted as above, and tracer influx into the brain was imaged ex vivo by a fluorescence microscope (Zeiss), with the tile function to rebuild the whole slice. Tracer influx figures were quantified independently by 2 sets of masked investigators using Fiji (ImageJ) software, as described in a previous study (3). Each coronal slice was manually outlined, and the mean fluorescence intensity was measured at a constant threshold. Average fluorescence intensity was calculated across 4 slices for a single animal. Equivalent slices were used for all biological replicates.

### Immunohistochemistry.

Mice were anesthetized with isoflurane and perfused transcardially with phosphate-buffered saline (PBS) followed by 4% paraformaldehyde. Brains were post-fixed overnight, followed by immersion in 30% sucrose for 24 hours. Coronal sections (40 μm) were cut on a microtome. Sections were immunostained with primary antibodies AQP4 (249323, 1:100, Alomone Labs), collagen IV (2150-1470, 1:100, Bio-Rad), and GFAP (13-0300, 1:100, Invitrogen, Thermo Fisher Scientific). Briefly, the sections were washed 3 times with PBS (10 minutes each time), then permeabilized by incubating with 0.3% Triton X-100 for 5 minutes, followed by incubation with blocking solution containing 3% donkey serum, 3% goat serum, and 0.3% Triton X-100 for 1 hour. The sections were then incubated with primary antibody at 4°C overnight. After 3 washes with PBS, the sections were incubated with fluorescence-labeled secondary antibody for 2 hours at room temperature and then washed 3 times with PBS. Sections were mounted onto Superfrost slides (Thermo Fisher Scientific), dried, and then covered with SlowFade Gold Antifade Mountant (Invitrogen). Fluorescence images were acquired with an LSM 700 Axio Observer fluorescence microscope. *Z*-stacks correspond to the emission spectrum (405 nm DAPI, 488 green, 555 red) (resolution was 1,024 × 1,024 pixels) (0.2 μm/px) on *x,y* axis and a *Z*-axis step of 1 μm.

For image analysis, the samples were coded with ID, and the images were analyzed by investigators masked to genotypes and patient information. Results were then calculated statistically and decoded by different investigators at the end. The results from 3 microscopy fields per slide and 3 sections per mouse were calculated for each mouse brain. The colocalization quantities (AQP4^+^collagen IV^+^) were determined using the analyze particles plugin function of ZEN3.4 (blue edition). The numbers of AQP4 and collagen IV–positive puncta in the field (160 × 160 μm^2^) were determined at a constant threshold for each stain using ×20 original magnification images for quantifications.

To count the number of astrocytes in the striatum, tiled images were captured using a Zeiss Axio Observer Z1 epifluorescence microscope. After the images were acquired, results were manually calculated statistically by different investigators. The whole striatum per slide and 3 sections per mouse were counted for each mouse’s brain. The number of astrocytes in the striatum was quantified at a constant threshold for each stain using ×20 original magnification images.

### Western blotting.

Striatal tissue samples were homogenized in a buffer containing 50 mM Tris-HCl at pH 8.0, 150 mM NaCl, 0.1% (w/v) SDS, 1.0% NP-40, 0.5% sodium deoxycholate, and 1% (v/v) protease inhibitor mixture. For SDS-PAGE, 20 μg of proteins were separated in a 4%–20% gradient gel and transferred to a nitrocellulose membrane. The membrane was blotted with the following primary antibodies: AQP4 (249323, 1:100, Alomone Labs), SNTA-1 (PA5-77702, 1:1,000, Thermo Fisher Scientific), GFAP (13-0300, 1:1,000, Thermo Fisher Scientific), GLUT1 (SPM498, 1:1,000, Thermo Fisher Scientific), GLUT3 (MA5-32697, 1:1,000, Thermo Fisher Scientific), and mouse anti–β-actin (MAB1501, MilliporeSigma, mouse monoclonal antibody, 1:5,000). After incubation with HRP-conjugated secondary antibodies, either goat anti-mouse IgG (H+L) (catalog 31430, Thermo Fisher Scientific) or goat anti-rabbit IgG (H+L) (catalog 31460, Thermo Fisher Scientific), bound antibodies were visualized by chemiluminescence. The intensity of the Western blot bands was quantified by ImageJ software.

### Statistics.

Data are expressed as the mean ± SEM unless otherwise noted. Student’s 1-tailed *t* test was used to measure the significant levels between WT or control and zQ175/+ or human HD groups at each given time point. *P* values less than 0.05 were considered statistically significant. Technical replication is represented by *n* in the figure legends.

### Study approval.

All animal experiments were performed in accordance with the *Guide for the Care and Use of Laboratory Animals* (National Academies Press, 2011) of the NIH and approved by the Institutional Animal Care and Use Committee at Johns Hopkins University.

### Data availability.

All data associated with this study are present in the paper or the supplement. Supporting data values associated with the main manuscript and supplement material, including values for all data points shown in graphs and values behind any reported means, were provided in a single Supporting Data Values Excel (XLS) file. Additional requests for raw and analyzed data or materials should be made to the corresponding authors and will be promptly reviewed to determine whether the application is subject to any intellectual property or confidentiality requirements. Any data and materials that can be shared will be released after the execution of a material transfer agreement.

## Author contributions

WD, H Liu, and JX conceptualized the study, designed/performed experiments, analyzed/interpreted results, and wrote the manuscript. L Chen, CZ, CL, YL, L Cheng, YO, CR, JA, ZW, and ZZ performed experiments. H Lu, PCMVZ, and JJI interpreted results, provided resources, and edited and finalized the manuscript.

## Supplementary Material

Supplemental data

Unedited blot and gel images

Supporting data values

## Version 1 09/03/2024

In-Press Preview

## Version 2 10/22/2024

Electronic publication
