# Supplementary material for: Glymphatic influx and clearance are perturbed in Huntington’s disease
Source: JCI Insight. 2024 Oct 22;9(20):e172286. doi: 10.1172/jci.insight.172286 (PMC11530125; doi:10.1172/jci.insight.172286)
Supplement: Supplemental data [file jciinsight-9-172286-s033.pdf]

Supplementary Materials

Materials and Methods

Animals

Aqp4 KO mouse breeders were kindly provided by Dr. Quigley's lab at Johns Hopkins University School of Medicine (Baltimore, MD, USA); the original line of *Aqp4* KO mice was generated by Dr. Ole Petter Ottersen using the Genoways technique as described previously (Thrane et al., 2011). Their strategy involved cloning and sequencing a targeted region of the murine *Aqp4* gene in a 129/Sv genetic background. Identification of a targeted locus of the *Aqp4* gene permitted the deletion of exons 1–3 to avoid any expression of putative splice variants. Hence, a flippase recognition target (FRT)-neomycin-FRT-LoxP–validated cassette was inserted downstream of exon 3 and a LoxP site was inserted upstream of exon 1 (Thrane et al., 2011). The mice were backcrossed for 20 + generations with C57BL/6N mice prior to experimentation.

In Vivo structural MRI acquisition

In vivo MRI was performed on a vertical bore 9.4 Tesla MR scanner (Bruker Biospin, Billerica, MA, USA) with a triple-axis gradient and a physiological monitoring system (EKG, respiration, and body temperature). Mice were anesthetized with isoflurane (1%) mixed with oxygen and air at 1:3 ratios via a vaporizer and facial mask and scanned longitudinally (the same mice were imaged repeatedly over 12 months). We used a 20-mm diameter volume coil as the radiofrequency transmitter and receiver. The temperature was maintained by a heating block built into the gradient system. Respiration was monitored throughout the entire scan.

High-resolution anatomical images were acquired using a three-dimensional (3D) T2-weighted fast spin echo sequence with the following parameters: echo time (TE)/repetition time (TR) = 40/700 ms, resolution = 0.1 mm × 0.1 mm × 0.25 mm, echo train length = 4, number of averages = 2, and flip angle = 40°. Multi-slice T2-weighted images of the mouse brain were acquired by the RARE (Rapid Acquisition

with Refocused Echoes) sequence with the following parameters (echo time (TE) / repetition time (TR) = 40 ms/1500 ms, RARE factor = 8, in-plane resolution = 0.125 mm x 0.125 mm, slice thickness = 1 mm, total imaging time less than 2 min) and used for high-resolution anatomical imaging. The total imaging time was about 50 min per mouse. Mice recovered quickly once the anesthesia was turned off, and all mice survived the imaging sessions.

Structural MRI image analysis

Images were first rigidly aligned to a template image using automated image registration software (http://bishopw.loni.ucla.edu/AIR5/, AIR). The template image was selected from one of the images acquired from age-matched littermate control mice (the mouse had the medium brain volume among the control group), which had been manually adjusted to the orientation defined by the Paxinos atlas with an isotropic resolution of 0.1 mm x 0.1 mm x 0.1 mm per pixel. After rigid alignment, images had the same position and orientation as the template image, and image resolution was also adjusted to an isotropic resolution of 0.1 mm × 0.1 mm × 0.1 mm per pixel. Signals from non-brain tissue were removed manually (skull-stripping). Skull-stripped, rigidly aligned images were analyzed by using Landmarker software (www.mristudio.org). Intensity values of the gray matter, white matter, and cerebral spinal fluid were normalized to the values in the template images using a piece-wise linear function. This procedure ensured that the subject image and template image had similar intensity histograms. The intensity-normalized images were submitted by Landmarker software to a Linux cluster, which runs Large Deformation Diffeomorphic Metric Mapping (LDDMM). The transformations were then used for quantitative measurement of changes in local tissue volume among different mouse brains by computing the Jacobian values of the transformations generated by LDDMM (Zhang et al., 2010). There are 29 different brain regions segmented automatically.

D-Glucose uptake image analysis

The average water signal intensity before infusion is S_{base}. Motion correction was conducted using Medical Imaging Registration Toolbox (Myronenko et al., 2010). The regions of interest (ROIs) were manually selected according to the mouse brain atlas (https://mouse.brain-map.org). The glucose uptake curves detected by ^{1}H MRS and DGE MRI were fitted by exponential functions to make a quantitative comparison (Huang et al):

$$\Delta S(t) = \frac{S_{base} - S(t)}{S_{base}} x100\% = \Delta S_{max}(t - t_0)^{\frac{1}{\mu_{in}}} \mathrm{e}^{-\mu_{out} \cdot (t - t_0)} \quad \text{[S1]}$$

Where $\Delta S(t)$ is the signal difference determined by DGE MRI. μ_{in} and μ_{out} are the glucose uptake and outflow rates, respectively. ΔS_{max} represents the maximum signal difference for DGE. A two-sample *t*-test was conducted on the fitted parameters between the HD and WT groups.

Colocalization density analysis

ZEN 3.4(Black edition) was used to do the Colocalization analysis. The threshold of CH1 is set to 80 to define the green signal (collagen IV). The threshold of CH2 is set to 120 to define the red signal (AQP4). In this way, the Collagen IV area (green pixels), the AQP4 area (red pixels) and the colocalized area (yellow pixels) are generated. Colocalization density was quantified as the percentage of collagen IV and AQP4 positive immunofluorescence within the collagen IV area (yellow pixels / green pixels).

Intra-striatal tracer injection

To study the efflux of the brain, mice were perfusion fixed 3 hours after intra-striatal tracer injection. 100µm coronal vibratome slices were cut and mounted as above, the whole slice was captured using a fluorescence microscope (Zeiss) with the tile function. Tracer efflux figures were quantified independently by two sets of blinded investigators using Fiji (Image J) software, as described in a previous study.

Behavioral tests

5mm balance beam testing was conducted on an 80 cm long and 5 mm wide square-shaped balance beam that was mounted on supports of 50 cm in height. A bright light illuminated the start platform, and a darkened enclosed 1728 cm^3 escape box (12 × 12 × 12 cm^3) was situated at the end of the beam. Mice were trained to walk across the beam twice at least 1 h prior to testing. The time for each mouse to traverse the balance beam was recorded with a 125-sec maximum cut-off, and falls were scored as 125 sec.

References (for Supplementary data only)

Thrane AS, Rappold PM, Fujita T, Torres ABekar LK, Takano T, Peng W, Wang F, Rangroo Thrane V, Enger R, Haj-Yasein NN, Skare Ø, Holen T, Klungland A, Ottersen OP, Nedergaard M, Nagelhus EA (2011) Critical role of aquaporin-4 (AQP4) in astrocytic Ca2+ signaling events elicited by cerebral edema PNAS 108:846–851.

Huang, J.; van Zijl, P. C. M.; Han, X.; Dong, C. M.; Cheng, G. W. Y.; Tse, K. H.; Knutsson, L.; Chen, L.; Lai, J. H. C.; Wu, E. X.; Xu, J.; Chan, K. W. Y., Altered d-glucose in brain parenchyma and cerebrospinal fluid of early Alzheimer's disease detected by dynamic glucose-enhanced MRI. *Sci Adv* **2020,** *6* (20), eaba3884.

Myronenko A, Song X. Intensity-based image registration by minimizing residual complexity. IEEE Trans Med Imaging 2010; 29: 1882–18-91.

Zhang J, Peng Q, Li Q, Jahanshad N, Hou Z, Jiang M, Masuda N, Langbehn DR, Miller MI, Mori S, Ross CA, Duan W. Longitudinal characterization of brain atrophy of a Huntington’s disease mouse model by automated morphological analyses of magnetic resonance images. Neuroimage, 2010, 49(3):2340-51.

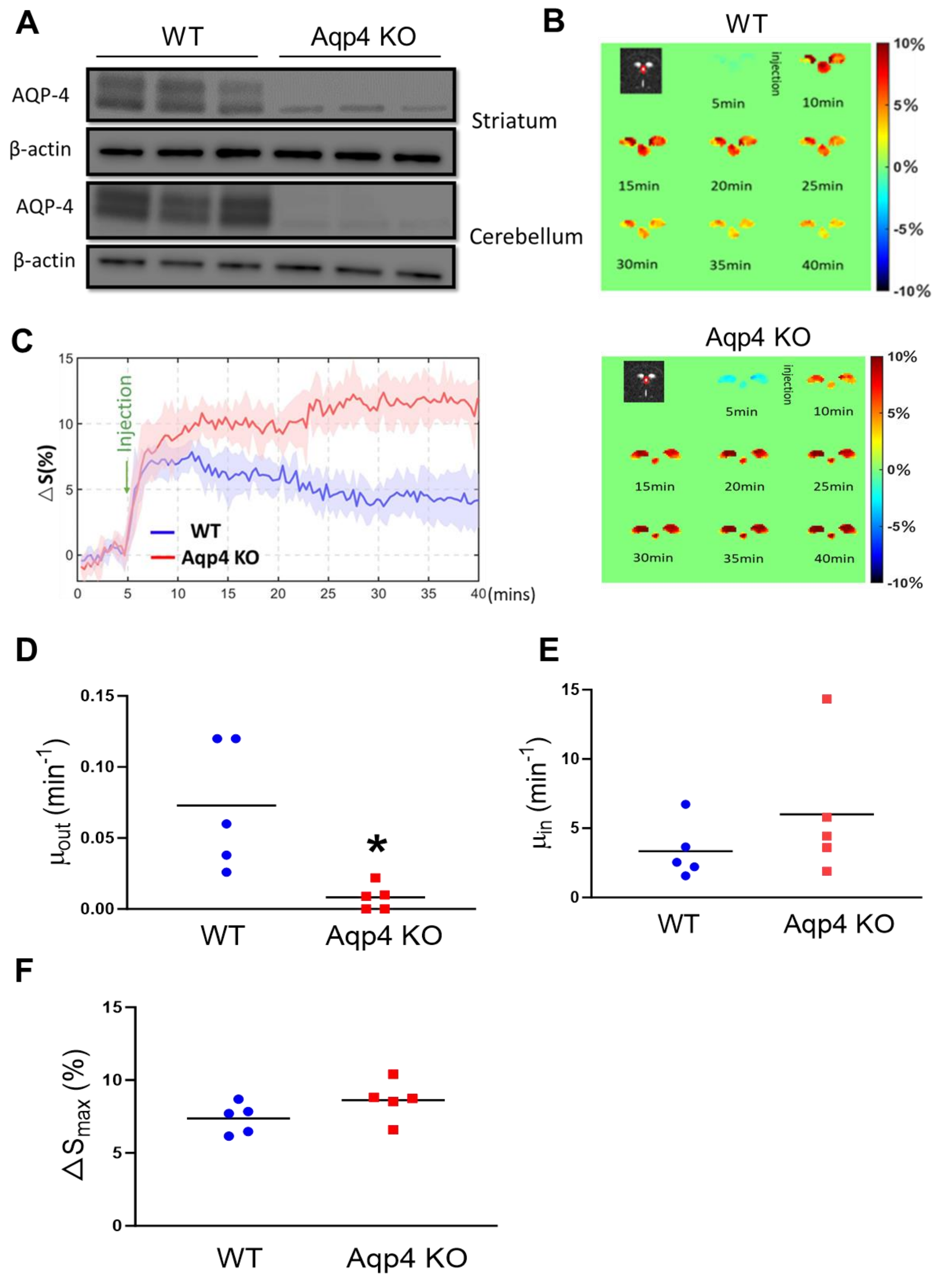

Figure S1. DGE MRI detects perturbed glymphatic function in Aqp4 KO mice at 4 months. (**A**) Western blots of AQP4 in the striatum and cerebellum of Aqp4 KO mice and wild-type (WT) controls. (**B**) Representative DGE images (signal described in Eq. S1) as a function of time in a WT mouse (upper panel) and an Aqp4 KO mouse (lower panel) at 4 months of age. (**C**) The average dynamic D-glucose signals in CSF during the entire scan period from WT (n=5) and Aqp4 KO (n=5) mice. (**D-F**) Comparison of fitted uptake parameter μ_{in} (D), clearance parameter μ_{out} (E) and maximal glucose levels ΔS_{max} (F) between WT and Aqp4 KO mice. *p<0.05 vs. WT by Standard Student's t-test.

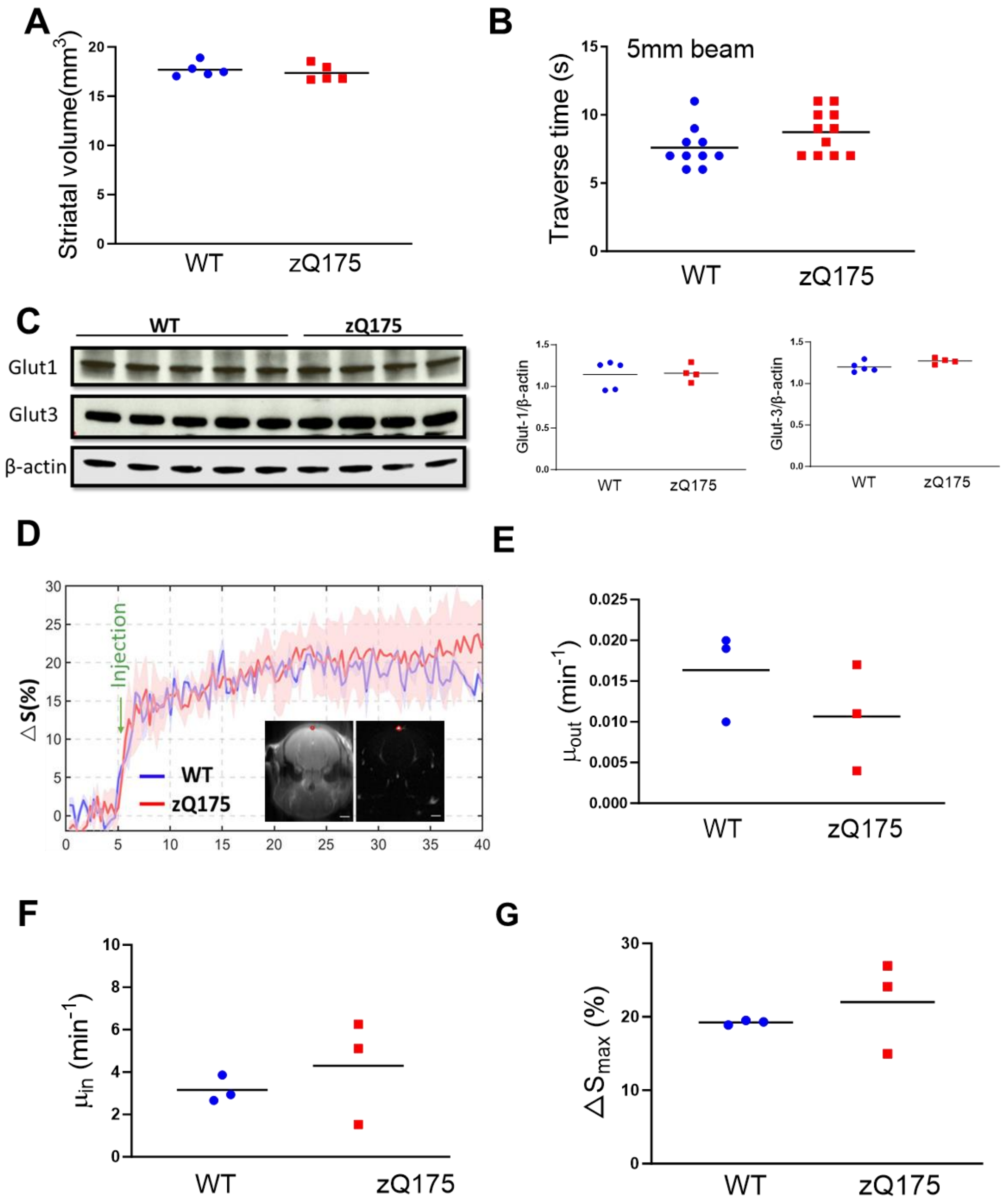

Figure S2. No difference in the striatal volume, motor function, and D-glucose uptake and clearance in the venous blood (sagittal sinus) between 4-month-old zQ175 and WT mice. (**A**) Striatal volume measured by structural MRI in zQ175 and age-matched WT mice. n=5 mice/group. (**B**) Motor coordination assessment on a 5mm balance beam, the traverse time was recorded from WT (n=9) and zQ175 (n=11) mice. (**C**) Western blots and quantification of glucose transporters Glut1 and Glut3 in the striatum of mice. (**D**) DGE curves for the sagittal sinus (red ROI) in WT and zQ175 mice. ROI within the blood vessel (red circle). (**E-G**) D-glucose clearance rate (E), uptake rate (F), and maximal glucose levels in the sagittal sinus vein following i.v injection of D-glucose in the tail vein (G).

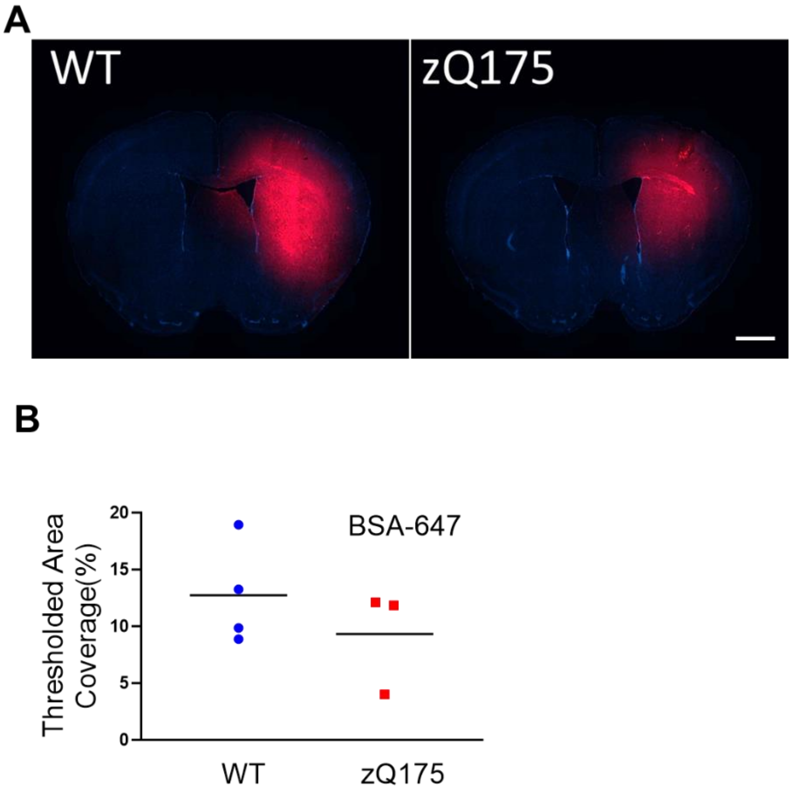

Figure S3. **Glymphatic efflux was examined in 4 months old zQ175 HD mice**. BSA-647 fluorescent probe was injected into the striatum of mice. The mice were perfused, and images were captured at 180 minutes after the probe injection. (**A**) Representative images from one wile type (WT) control mouse and one zQ175 HD mouse. Red fluorescence indicates the BSA-647 dye, blue fluorescence is DAPI staining. Scale bar = 1cm. (**B**) Quantitation of BSA-647 fluorescent area. Male mice, n= 4 WT and 3 HD.

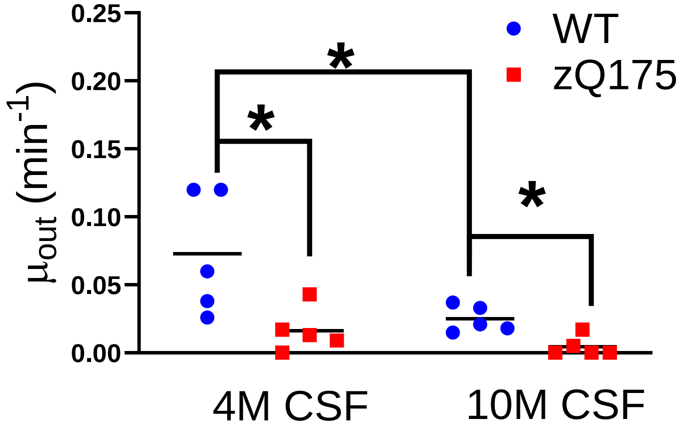

Figure S4. **Comparison of D-glucose clearance speed in different ages of zQ175 and wild type control mice.** An age-dependent decline of the D-glucose clearance speed in wild type mice was noticed.

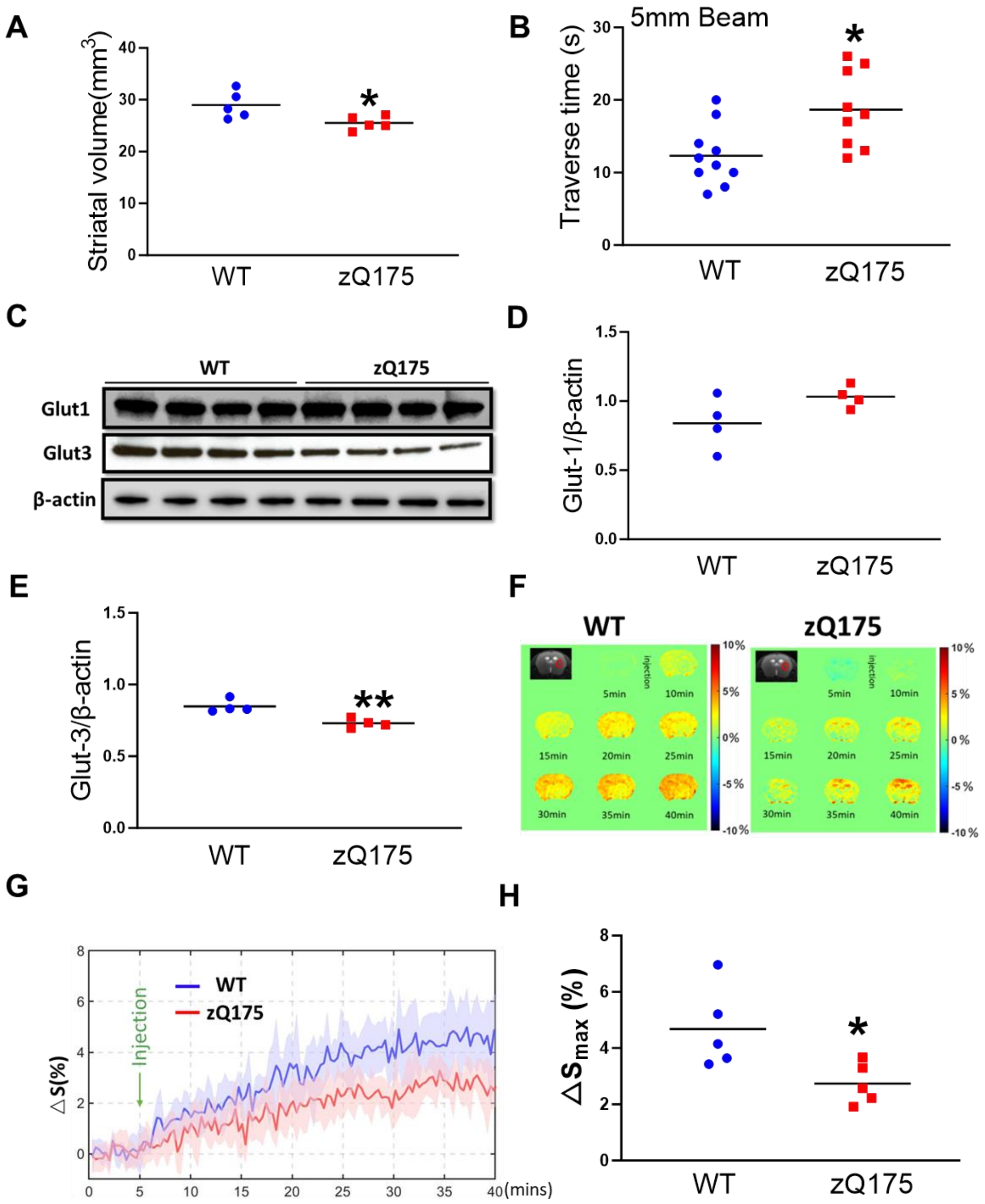

Figure S5. Reduced striatal volume, motor deficits, and perturbed D-glucose uptake in the striatum are evident in 10-month-old zQ175 mice. (**A**) Striatal volume measured by structural MRI in zQ175 and age-matched WT mice. n=5 mice/group. **p*<0.05 vs WT by Standard Student's t-test. (**B**) Motor coordination assessment on a 5mm balance beam, the traverse time was recorded from WT and zQ175 mice. n=10, **p*<0.05 vs. WT by Standard Student's t-test. (**C**) Western blots of Glut1 and Glut3 in the striatum of WT and zQ175 mice. (**D-E**) Quantification of Glut1 and Glut3 levels from Western blots in C. n=4, ***p*<0.01 vs. WT by Standard Student's t-test. (**F**) Representative images of D-glucose uptake in the striatum of WT and zQ175 mice. The hot color indicates higher uptake. (G) The DGE curves of D-glucose uptake following i.v. injection in the striatum of WT and zQ175 mice. n=5. Note decreased D-glucose uptake in HD mouse striatum. (**H**) The maximal D-glucose concentrations in WT and HD mouse striatum after i.v. infusion. n=5, **p*<0.05 vs. WT by Standard Student's t-test.

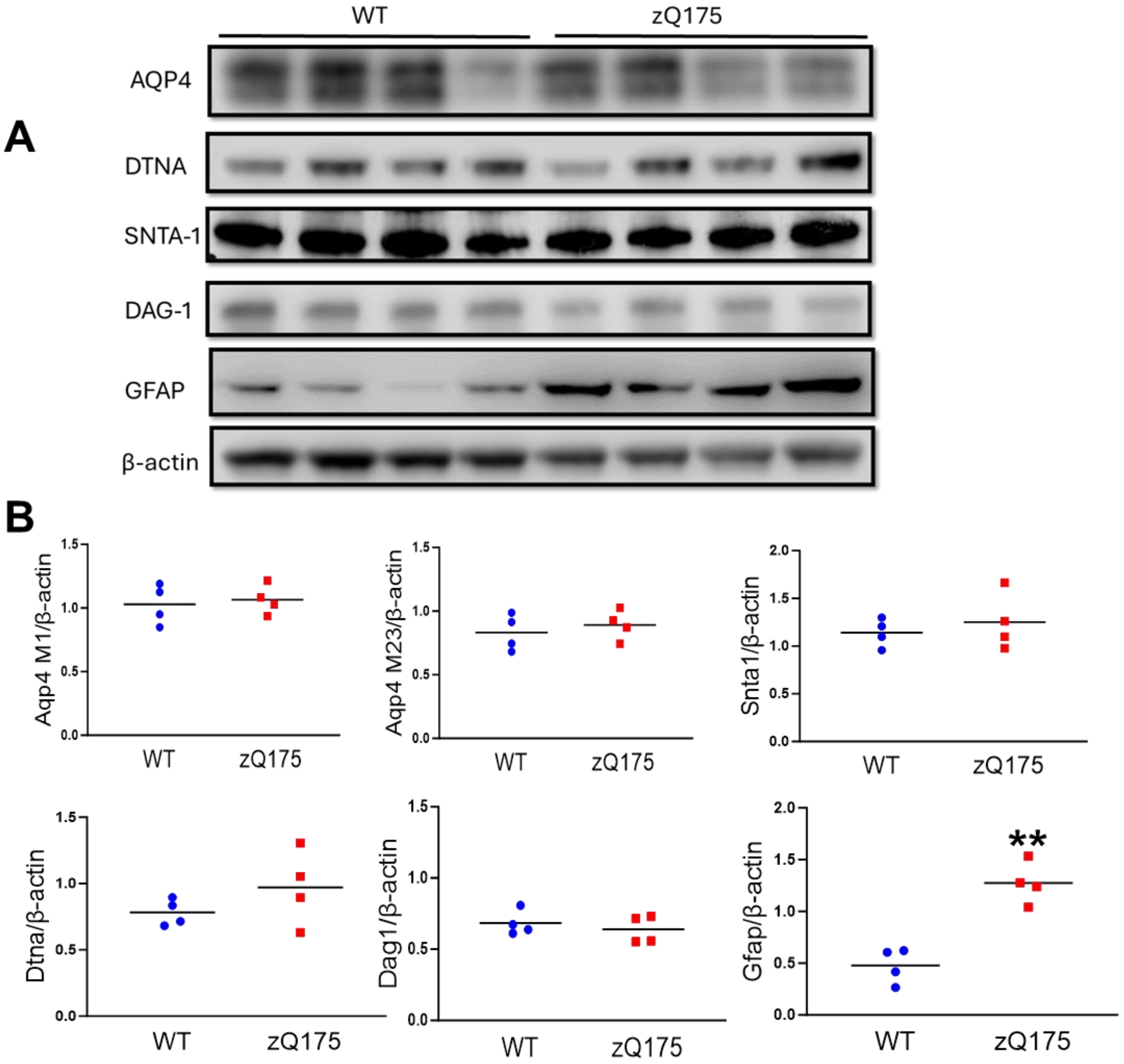

Figure S6. Astrogliosis in manifest zQ175 HD mice. (A) Western blots images of samples from 10-month-old mice. (**B**) Quantification of AQP4, SNTA1, DTNA, DAG1, GFAP in the striatum of 10-month-old zQ175 mice and WT controls. **p<0.01 vs. WT by Standard Student's t-test.

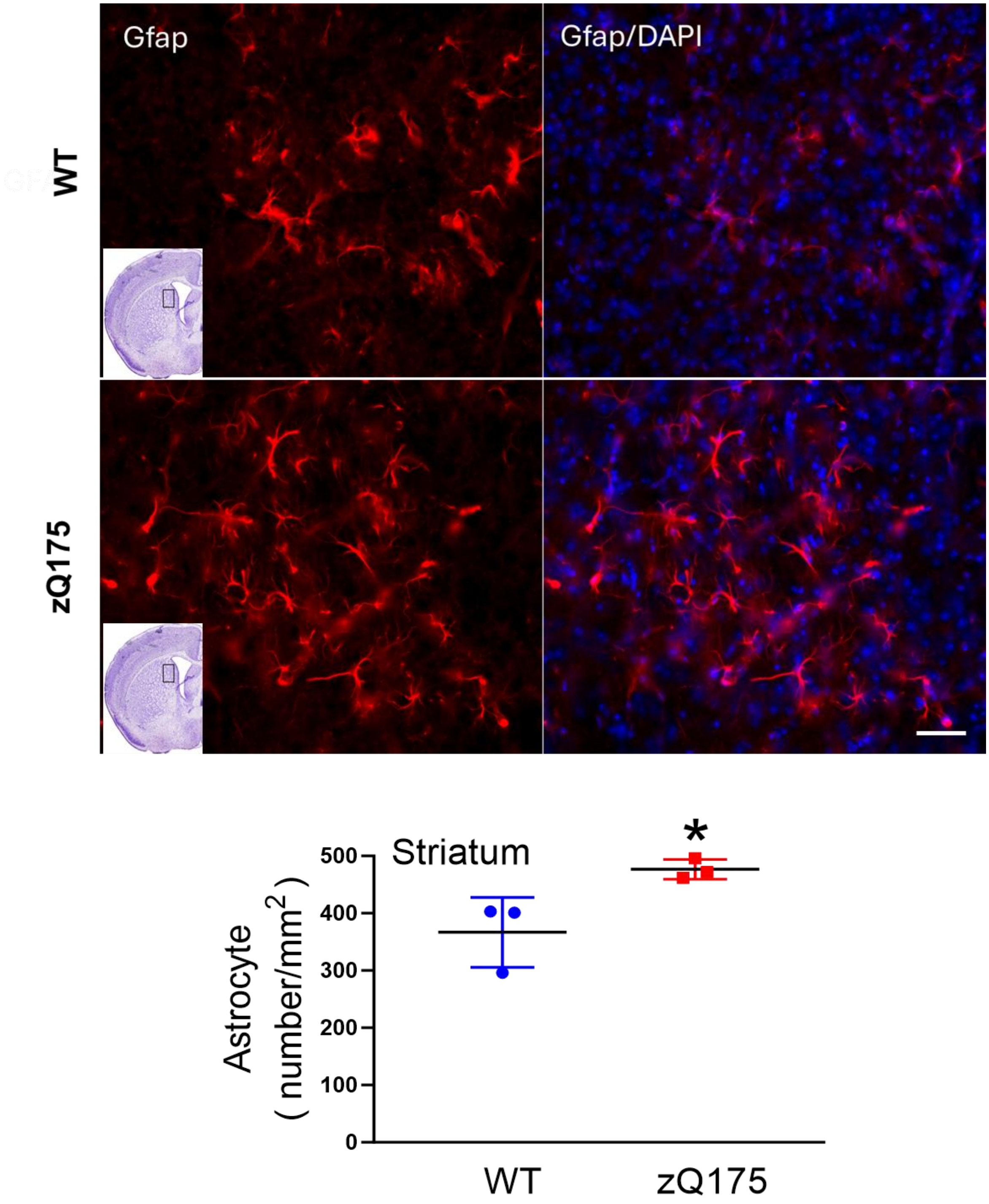

Figure S7. Astrogliosis was evident in the striatum of 10-month-old zQ175 mice. (**A**) Representative Gfap immunostaining in the striatum of zQ175 and wild type controls. Scale bar = 50 µm. (**B**) Quantification of numbers of Gfap positive cells per square mm. *p<0.05 vs the values of wild type controls by Standard Student's *t*-test. n=3 male mice/genotype.

Table S1. Postmortem caudate putamen samples from NIH Neurobiobank for immunostaining

Subject ID	Age	Brain Region	Subject Sex	Clinical Brain Diagnosis	Preparation
S04230	80	Caudate nucleus	Male	Huntington's disease	4% Paraformaldehyde Fixed
S08214	93	Caudate nucleus	Male	Huntington's disease	4% Paraformaldehyde Fixed
S08434	70	Caudate nucleus	Male	Unaffected Control	4% Paraformaldehyde Fixed
S09101	72	Caudate nucleus	Male	Huntington's disease	4% Paraformaldehyde Fixed
S13047	72	Caudate nucleus	Male	Huntington's disease	4% Paraformaldehyde Fixed
S16578	71	Caudate nucleus	Male	Unaffected Control	4% Paraformaldehyde Fixed
S16640	70	Caudate nucleus	Male	Unaffected Control	4% Paraformaldehyde Fixed
S16891	71	Caudate nucleus	Male	Unaffected Control	4% Paraformaldehyde Fixed
S18965	69	Caudate nucleus	Male	Unaffected Control	4% Paraformaldehyde Fixed

Table S2. Postmortem caudate putamen samples from NIH Neurobiobank for Western blotting

Subject ID	Age	Brain Region	Subject Sex	Clinical Brain Diagnosis	Preparation
70468	64	Caudate nucleus	Male	Unaffected Control	Frozen
90596	66	Caudate nucleus	Male	Unaffected Control	Frozen
4735	73	Caudate nucleus	Male	Unaffected Control	Frozen
8926	65	Caudate nucleus	Male	Unaffected Control	Frozen
Hct15HAU	65	Caudate nucleus	Male	Unaffected Control	Frozen
47453	55	Caudate nucleus	Male	Unaffected Control	Frozen
70468	64	Caudate nucleus	Male	Unaffected Control	Frozen
4494	67	Caudate nucleus	Male	Unaffected Control	Frozen
13232	65	Caudate nucleus	Male	Unaffected Control	Frozen
S07953	71	Caudate nucleus	Male	Huntington Grade 2	Frozen
S18494	71	Caudate nucleus	Male	Huntington Grade 2	Frozen
S09931	75	Caudate nucleus	Male	Huntington Grade 2	Frozen
S06621	75	Caudate nucleus	Male	Huntington Grade 2	Frozen
S13047	72	Caudate nucleus	Male	Huntington Grade 2	Frozen
S17130	76	Caudate nucleus	Male	Huntington Grade 2	Frozen
S19144	73	Caudate nucleus	Male	Huntington Grade 3	Frozen
S01799	71	Caudate nucleus	Male	Huntington Grade 3	Frozen
S09310	70	Caudate nucleus	Male	Huntington Grade 3	Frozen
S03632	75	Caudate nucleus	Male	Huntington Grade 3	Frozen
S16620	71	Caudate nucleus	Male	Huntington Grade 3	Frozen
S13520	71	Caudate nucleus	Male	Huntington Grade 3	Frozen
S19813	70	Caudate nucleus	Male	Huntington Grade 3	Frozen
