# Supplementary material for: Glymphatic influx and clearance are perturbed in Huntington’s disease
Source: JCI Insight. 2024 Oct 22;9(20):e172286. doi: 10.1172/jci.insight.172286 (PMC11530125; doi:10.1172/jci.insight.172286)

Figure 2E unedited images

4M AQP4

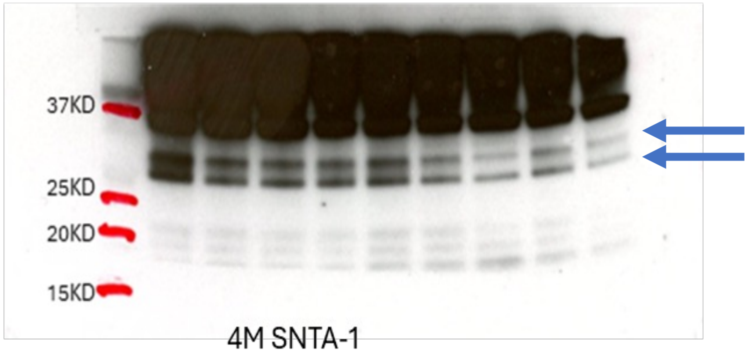

4M DAG-1

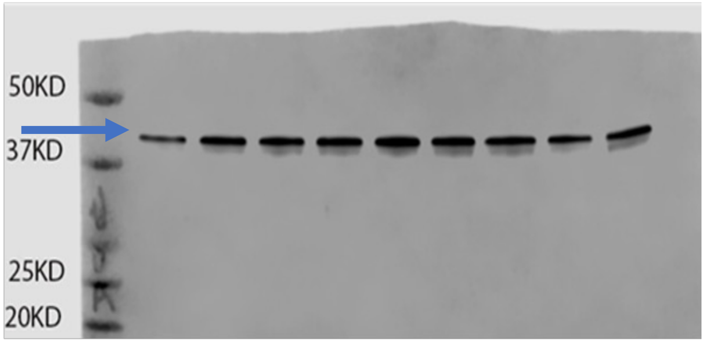

4M SNTA-1

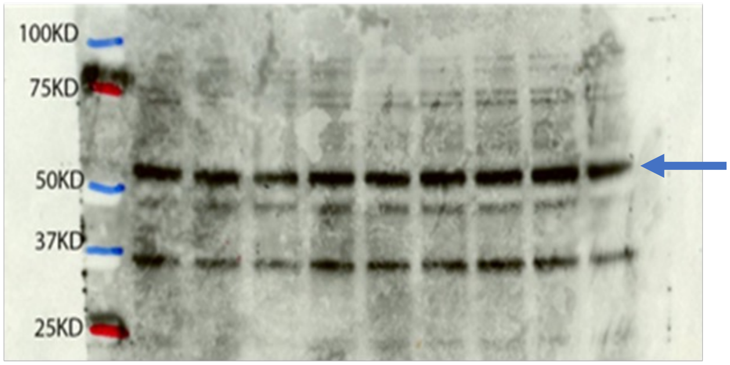

4M GFAP

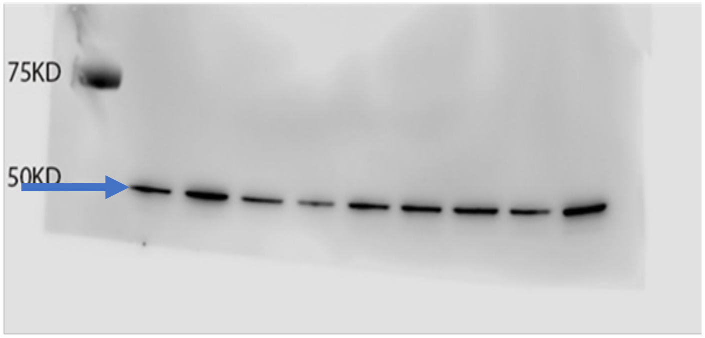

4M DTNA

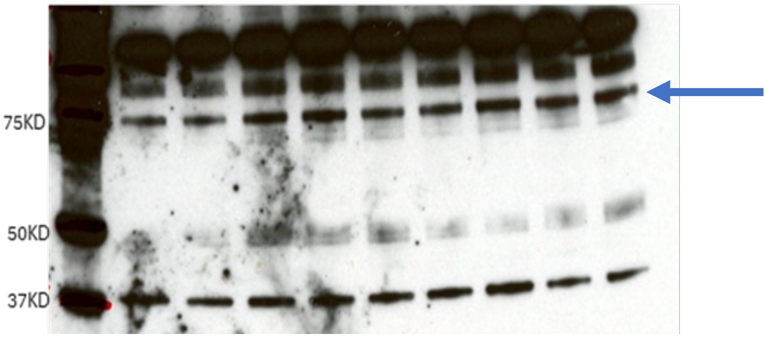

4M β-actin

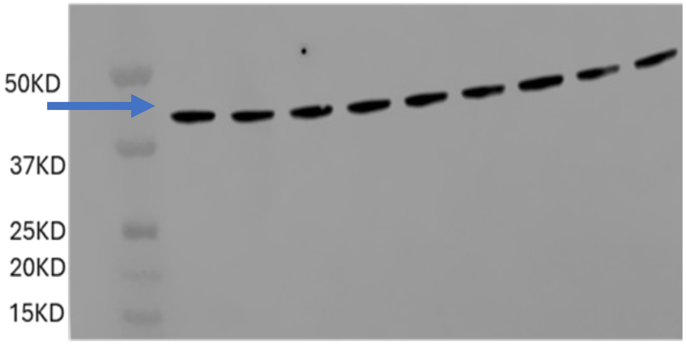

Figure 4C upper panel unedited images

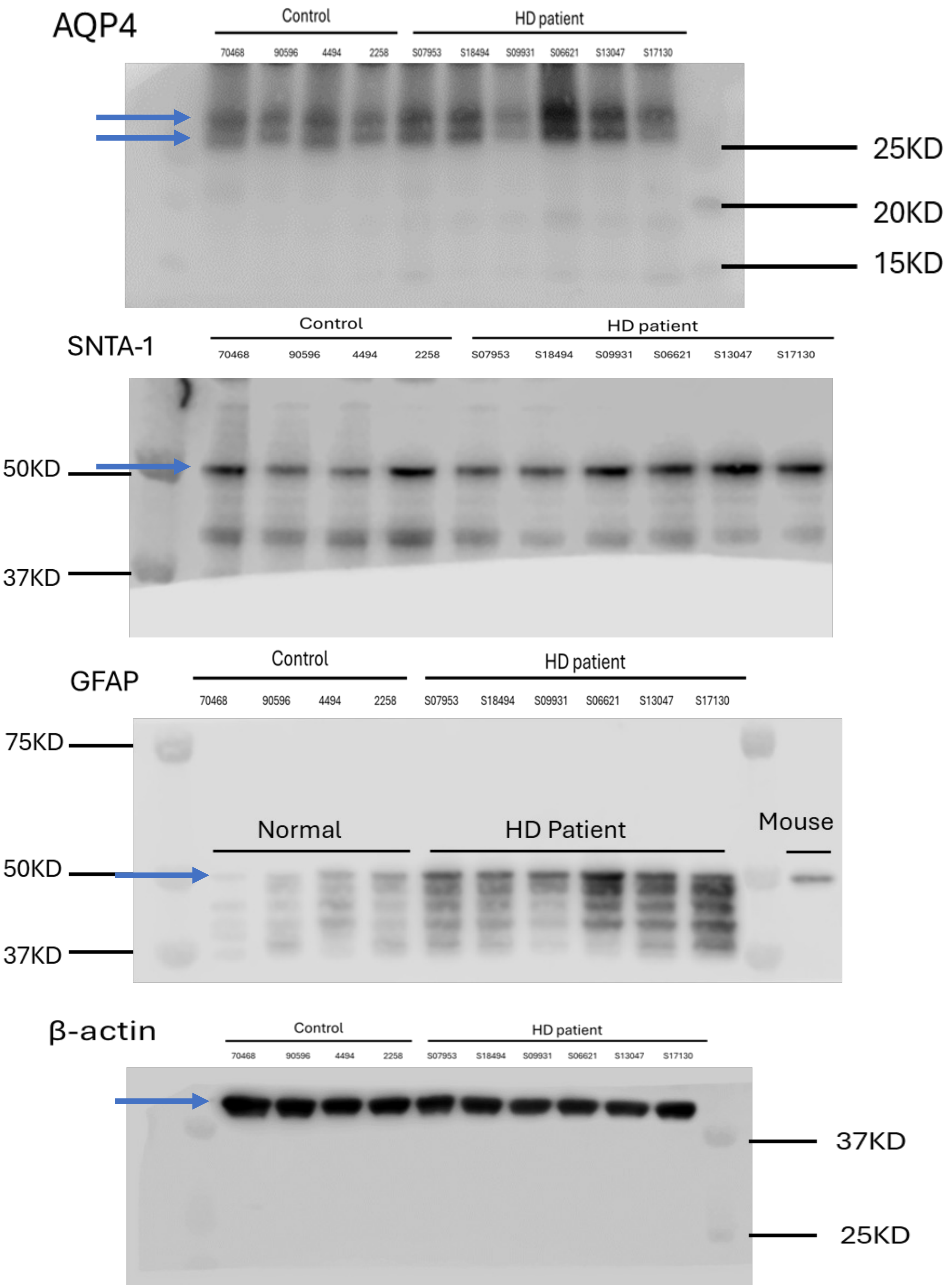

Figure 4C lower panel unedited images

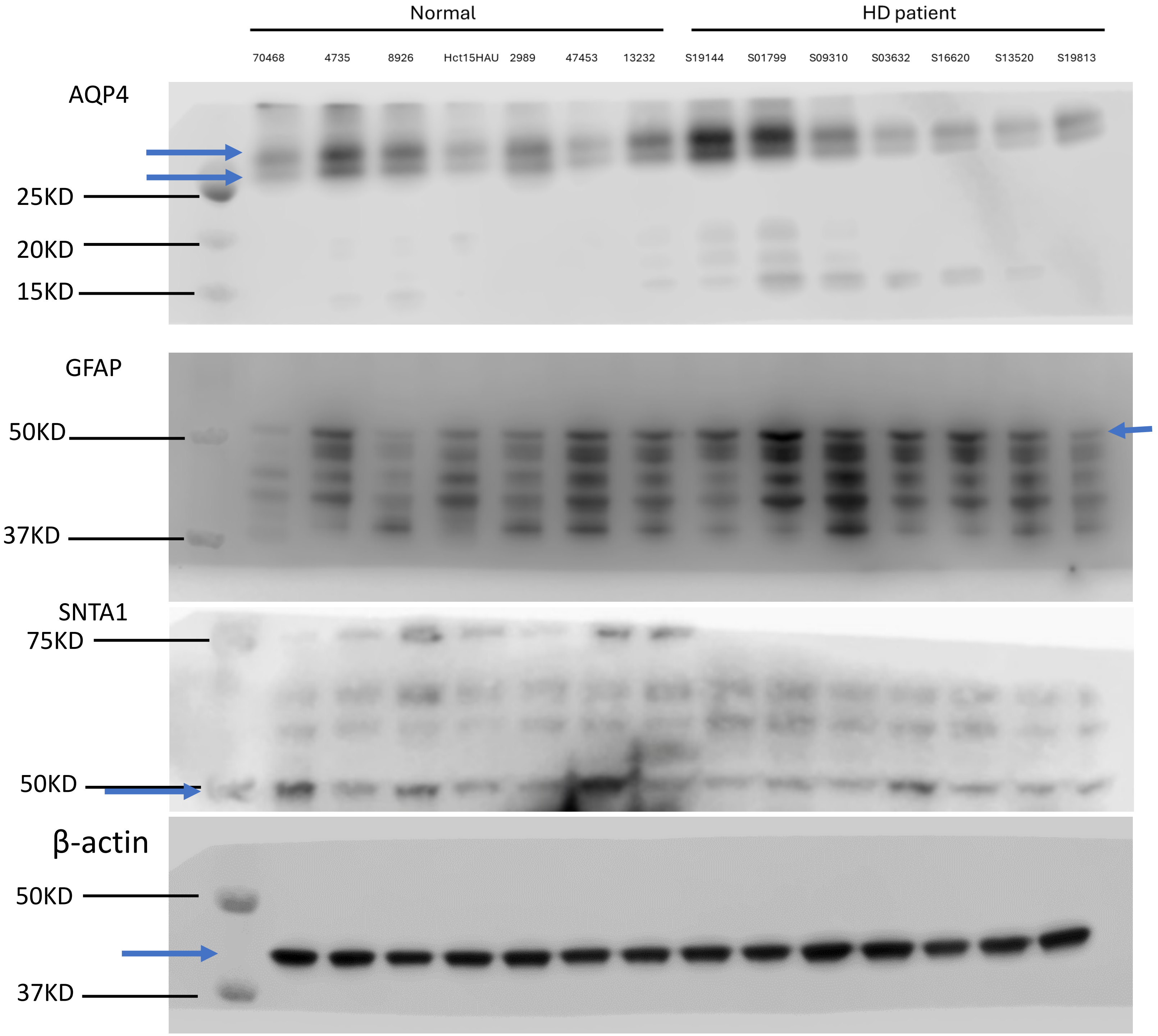

Figure S1A unedited images

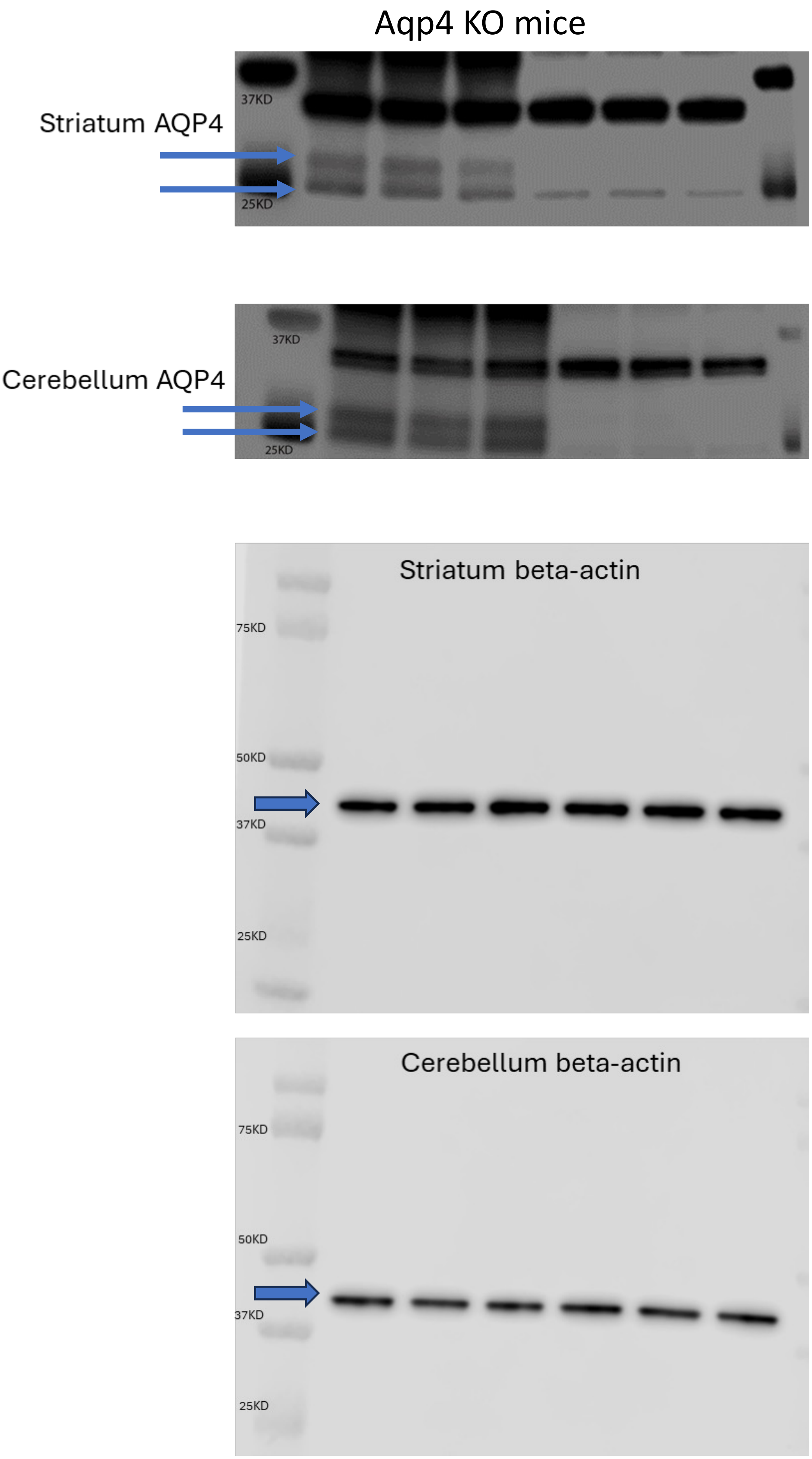

Figure S2C unedited images

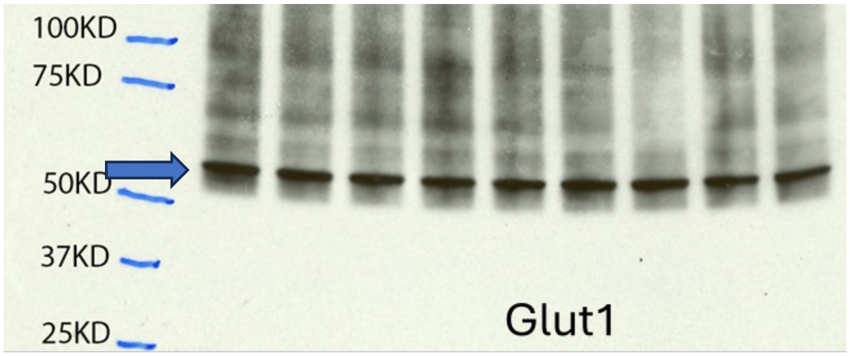

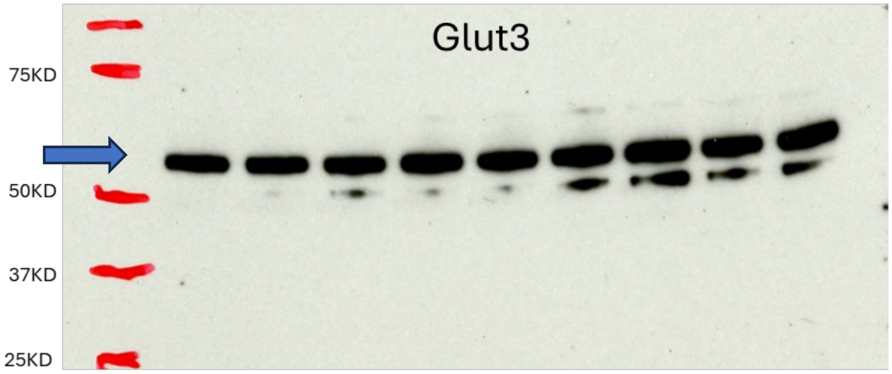

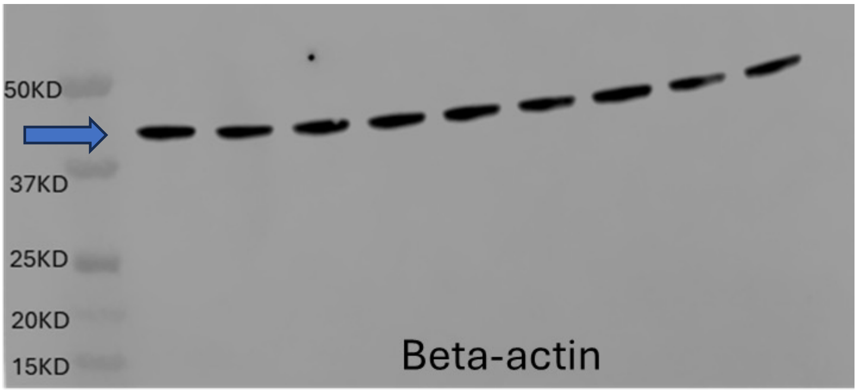

Figure S5C unedited images

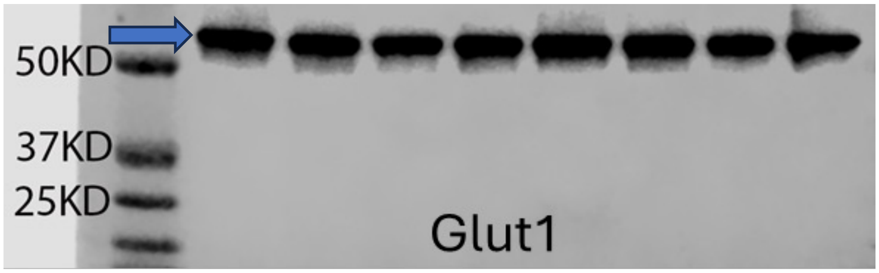

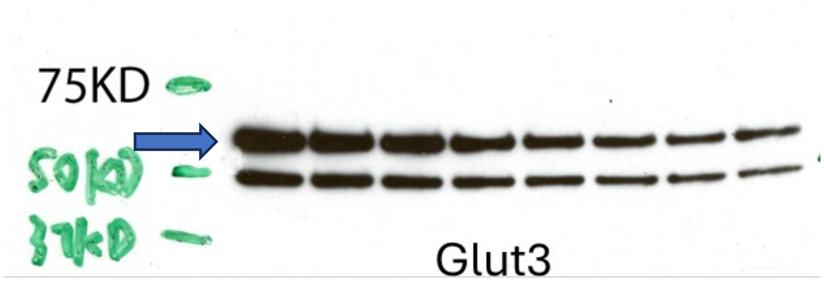

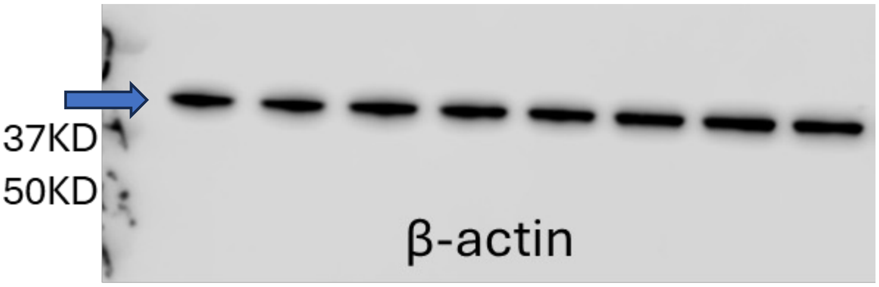

Figure S6A unedited images

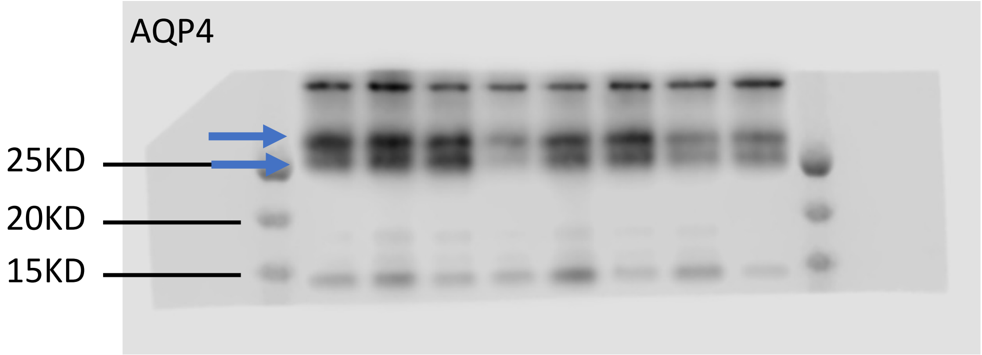

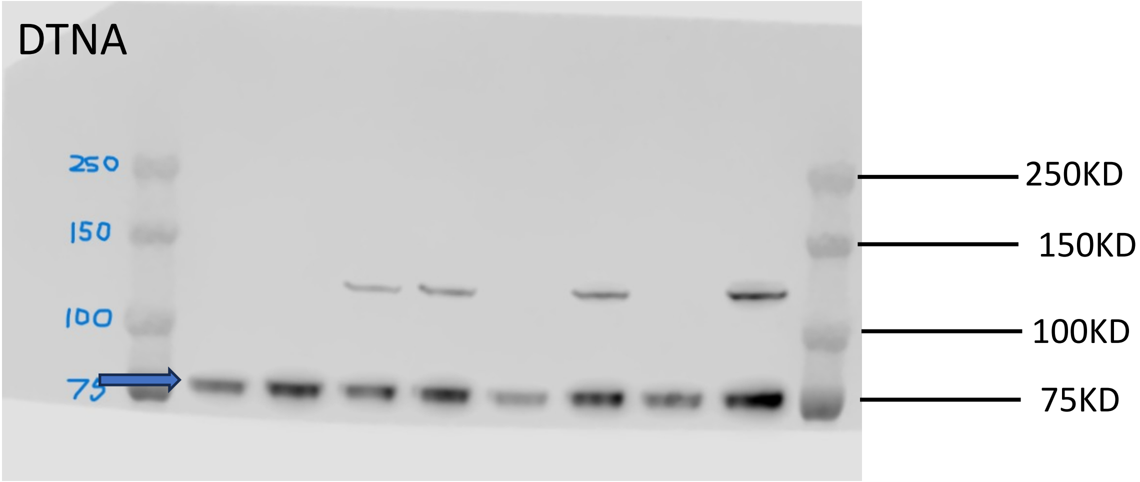

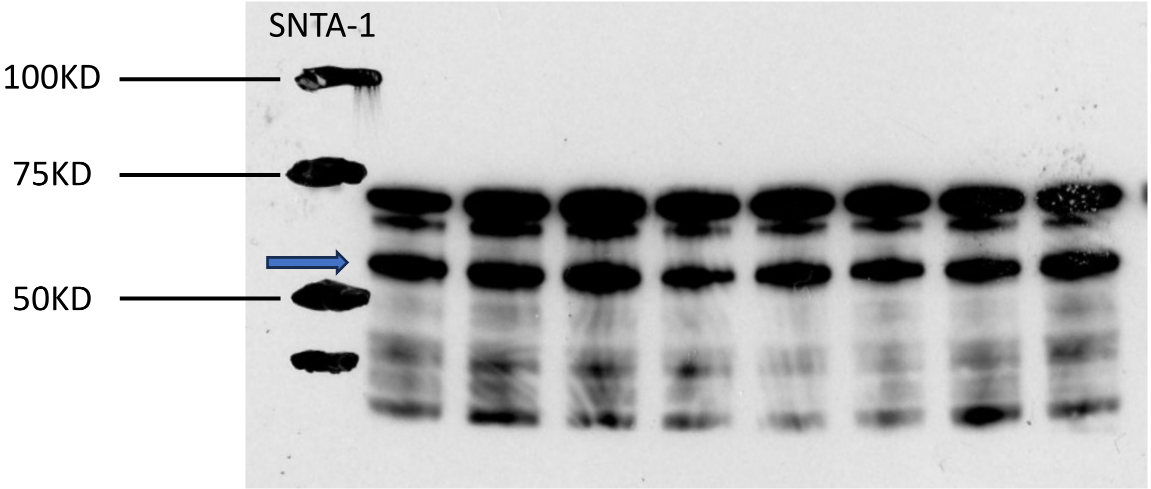

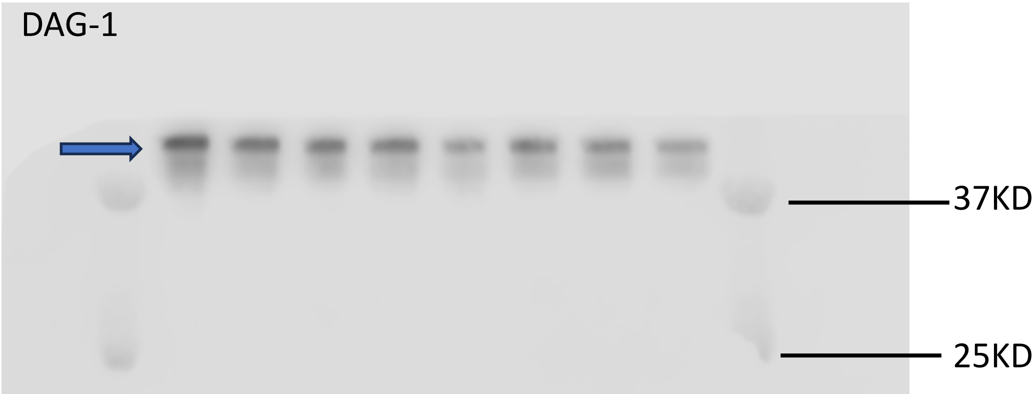

Figure S6A unedited images

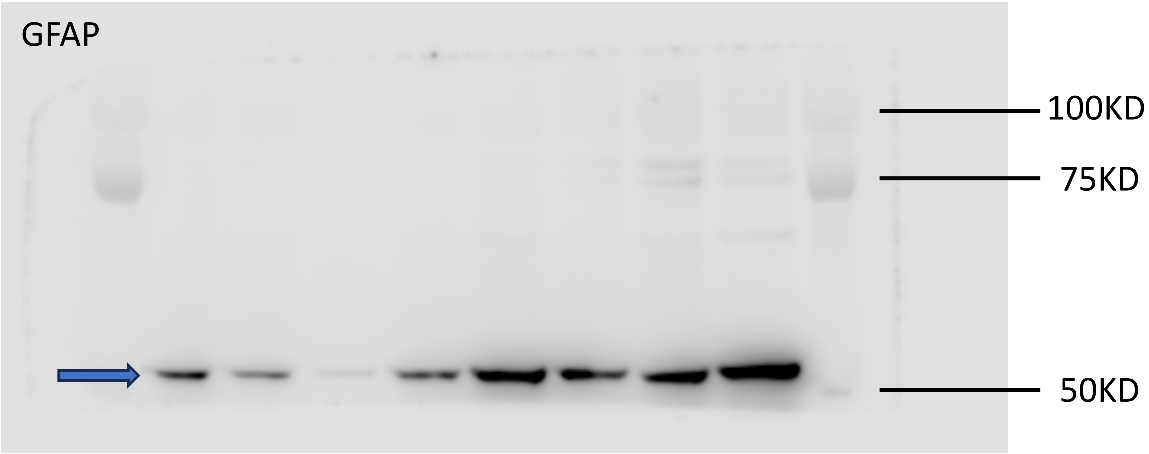

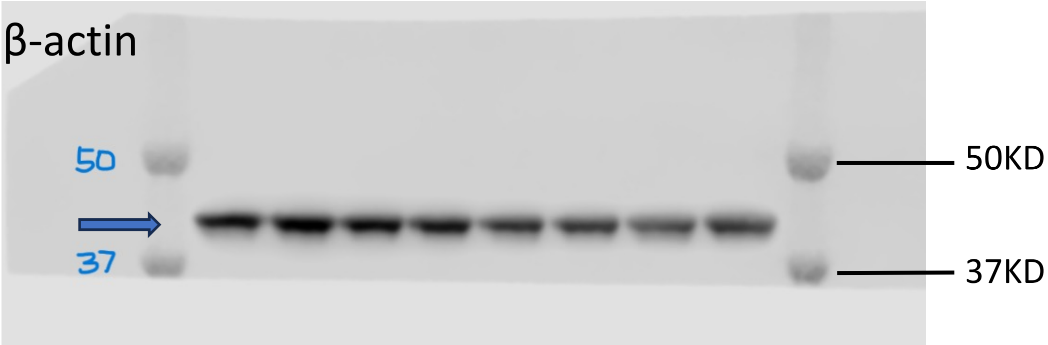

Supplement: Unedited blot and gel images [file jciinsight-9-172286-s034.pdf]
